# Unveiling the Unprecedented Optical Properties of Citrate‐Stabilized Hollow AgAu Nanoshells Under Photothermal Irradiation

**DOI:** 10.1002/smsc.202500494

**Published:** 2025-12-04

**Authors:** Gregory Q. Wallace, Jennifer Gracie, Amritpal Singh, Benjamin Clark, Kellie Jenkinson, Sara Bals, W. Ewen Smith, Tell Tuttle, Karen Faulds, Duncan Graham

**Affiliations:** ^1^ Department of Pure and Applied Chemistry Technology and Innovation Centre University of Strathclyde 99 George Street Glasgow G1 1RD UK; ^2^ Department of Pure and Applied Chemistry University of Strathclyde 295 Cathedral Street Glasgow G1 1XL UK; ^3^ Research Group EMAT Department of Physics, and Nanolab Centre of Excellence University of Antwerp Groenenborgerlaan 171 2020 Antwerp Belgium; ^4^ Present address: School of Chemistry University of Edinburgh Joseph Black Building, David Brewster Rd Edinburgh EH9 3FJ UK

**Keywords:** citrate, extinction spectroscopy, hollow gold nanoshells, photothermal irradiation, plasmonics, silver, surface‐enhanced Raman scattering

## Abstract

Metallic nanoshells heat efficiently on excitation of the localized surface plasmon resonance (LSPR). Whilst investigating the photothermal properties of citrate‐stabilized hollow gold nanoshells (HGNs) synthesized using a sacrificial silver nanoparticle (AgNPs), the LSPR undergoes a distinct blueshift (70 ± 20 nm (0.20 ± 0.06 eV)) when photothermally irradiated. Notably, when functionalized with a Raman reporter, the surface‐enhanced Raman scattering (SERS) signal unexpectedly and dramatically increases 8 ± 2‐fold upon plasmonic heating, despite the LSPR shifting away from the excitation wavelength. This unprecedented enhancement of the SERS signal is absent in samples lacking citrate or prepared using a cobalt nanoparticle template, underscoring the importance of citrate, heat, and AgNPs in eliciting these effects. It is hypothesized that aqueous silver ions near the surface of the HGNs react with the citrate and form a complex that is both light and temperature sensitive. The formation of silver deposits, observed by electron microscopy, alters the core‐to‐shell thickness ratio, resulting in a blueshift in the LSPR, and change the scattering to absorption properties, enabling an improved SERS performance. This new optical phenomenon has now been understood and will be of significant interest to future studies in harnessing the properties of HGNs.

## Graphical abstract

When photothermally irradiated, hollow gold nanoshells prepared using silver nanoparticles as templates and stabilized by citrate, exhibit unexpected optical changes. The localized surface plasmon resonance is blueshifts, and more surprisingly, the surface‐enhanced Raman scattering intensity of an adsorbed analyte increases. In the absence of either silver, citrate, or heat, this phenomenon is not observed.

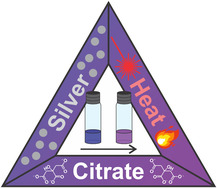

## 1 Introduction

Heating of metallic nanoparticles by localized surface plasmon resonance (LSPR) excitation is the basis of plasmonic photothermal therapy,^[^ ^1^, ^2^ ^]^ which has emerged as a treatment for cancers by creating localized hyperthermic environments or causing cell ablation, resulting in cell death. Many nanostructures and materials have been used for photothermal studies,^[^ ^3^, ^4^, ^5^, ^6^, ^7^, ^8^ ^]^ with metallic nanoshells, which support an LSPR in the near‐infrared (NIR) spectral range being a popular choice.^[^ ^9^, ^10^, ^11^ ^]^ For these types of structures, a thin metallic shell, typically gold, is grown around a solid particle, such as silica,^[^ ^12^ ^]^ or polystyrene.^[^ ^13^ ^]^ The optical properties of such structures are dependent on the core‐to‐shell size ratio. For the same sized core, a thinner shell exhibits a redshifted LSPR compared to a thicker shell.^[^ ^12^ ^]^ As the name implies, hollow metallic nanoshells have a hollow interior, due to the use of a sacrificial template as the inner core. These particles can be produced using a silica core and subsequent exposure to dilute hydrofluoric acid,^[^ ^14^, ^15^ ^]^ or with a metallic nanoparticle, where the metals are subsequently substituted by galvanic replacement.^[^ ^16^ ^]^

Cobalt nanoparticles (CoNPs) are commonly used templates for this approach.^[^ ^17^, ^18^, ^19^, ^20^ ^]^ However, the synthesis of CoNPs and galvanic replacement requires that the reaction be performed under inert conditions. The hollow interior forms once the sample is exposed to air. As such, when investigating optical properties of hollow metallic nanoshells by this method, it is often necessary to prepare new batches of CoNPs to evaluate different galvanic replacement ratios. Therefore, using alternative sacrificial nanoparticles that can be synthesized in large batches under ambient conditions is advantageous, as the same batch of nanoparticles can be used to evaluate different experimental parameters. In this regard, silver nanoparticles (AgNPs) have found promise as templates.^[^ ^21^, ^22^ ^]^ For both cobalt and silver, galvanic replacement with gold is commonly performed. The term hollow gold nanoshells (HGNs) is used although the resulting shell is an alloy of both the sacrificial and replacement metals. A potential advantage of using such structures compared to conventional nanoshells is that smaller, sub‐100 nm diameter particles can be synthesized, aiding entry into biological cells.^[^ ^23^ ^]^

This work seeks to understand a series of unprecedented results that were repeatedly observed for HGNs prepared by the galvanic replacement of AgNPs. When the HGNs were dispersed in a citrate solution, upon illumination, there was a distinct blueshift and change in extinction intensity of the LSPR. When the structure was functionalized with a Raman reporter, the blueshift was coupled with a multiple‐fold increase in the surface‐enhanced Raman scattering (SERS) intensity. Counter to expectations, this occurred even when the LSPR blueshifted further away from the 785 nm excitation used for the SERS measurements. Through a series of investigations, it was determined that three components played a vital role in the observed phenomena: the presence of citrate to help stabilize the HGNs, the temperature increase from photothermal irradiation, and the use of AgNPs as the sacrificial template as opposed to CoNPs. In the absence of citrate or when substituting CoNPs for AgNPs, these effects were not observed. We propose that Ag^+^ ions generated during the HGN formation are reduced to Ag^0^ by citrate and heat in a manner analogous to that used to make silver colloid, thus making the HGN surface silver rich and producing a blueshift in the LSPR. The formation of this Ag^0^ enriched surface, in turn, increases the scattering properties, resulting in the counterintuitive increase in the SERS signal as the LSPR shifts away from the NIR excitation.

## 2 Results and Discussion

### 2.1 Characterization and Photothermal Properties of Citrate‐Stabilized, Silver‐Template Hollow Gold Nanoshells

**Figure 1:**
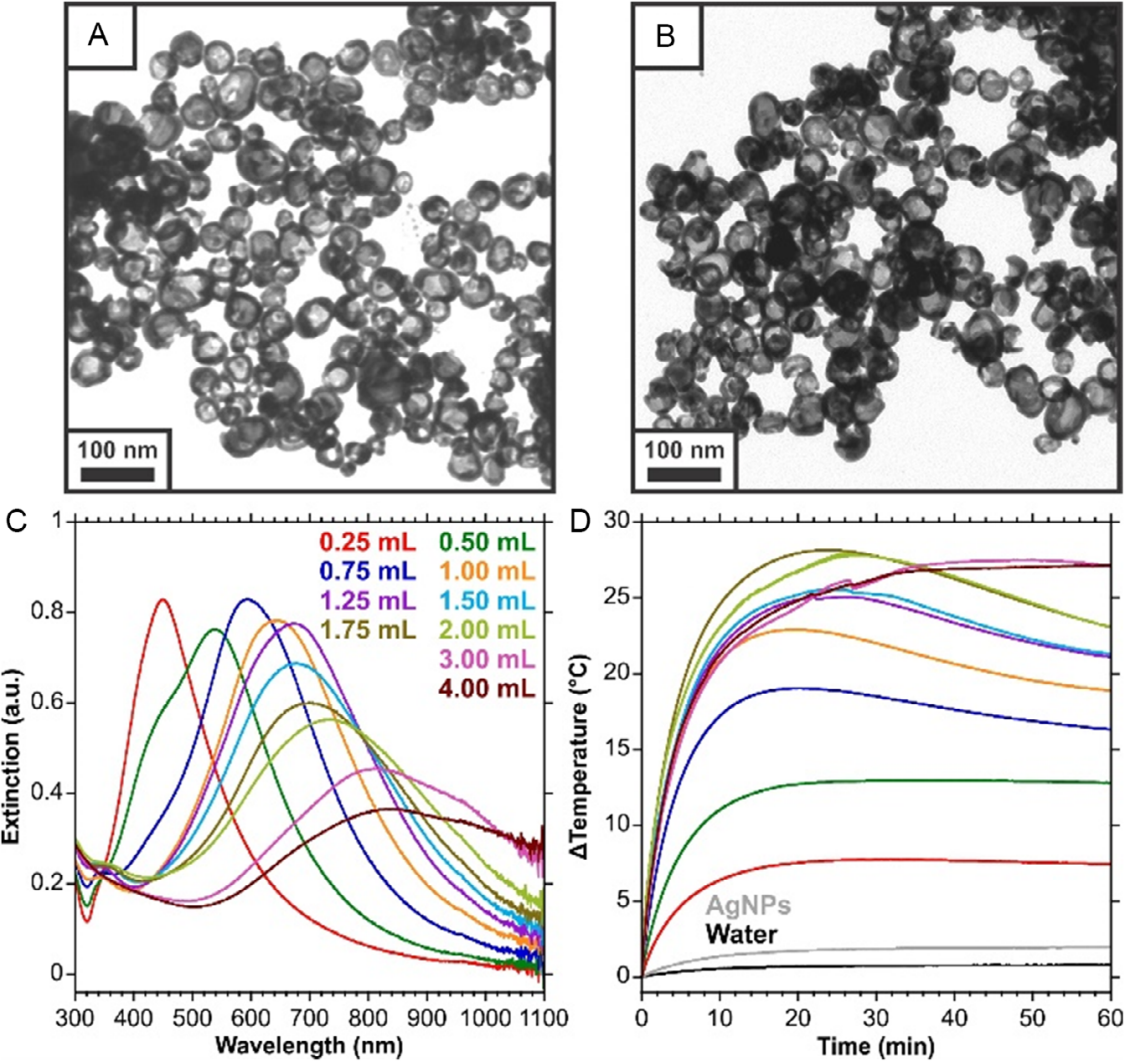
A) and B) TEM images of HGNs prepared with a 3:6:1 ratio of AgNPs: ddH_2_O: K/Au, equivalent to adding 1.00 mL of K/Au per 3.00 mL of AgNPs. C) Extinction spectra of HGNs prepared with different volumes of K/Au. D) Change in temperature profiles for those samples under 785 nm (280 mW) irradiation for 60 min.

The synthesis of HGNs made with AgNPs as the sacrificial template relies on the galvanic replacement of Ag^0^ with Au^0^ by AuCl_4_^−^.^[^ ^24^ ^]^ Details for the synthesis can be found in the supplementary information. Transmission electron microscopy (TEM) images of the prepared HGNs (**Figure** **1** A,B) show that the structures have a high degree of heterogeneity, which is consistent with galvanic replacement, as it is known that different shapes will form depending on the degree of galvanic replacement.^[^ ^25^ ^]^ This includes the presence of HGNs, nanorings, nanocages, etc.^[^ ^26^ ^]^ Consistent with the results that others have obtained,^[^ ^21^, ^26^, ^27^, ^28^, ^29^, ^30^, ^31^, ^32^, ^33^ ^]^ increasing the amount of K_2_CO_3_/HAuCl_4_ (K/Au) added increases the amount of galvanic replacement, leading to a shift in the position of the LSPR from the visible through to the NIR (Figure 1C). The ratios used are provided in Table S1, Supporting Information. The shift in the LSPR is accompanied by observable color differences between the samples (Figure S1, Supporting Information).

As Figure 1D demonstrates, the HGNs that have LSPRs in the NIR and have a higher extinction at 785 nm achieve the highest and fastest increases in bulk solution temperature following laser irradiation. Here, the same volume of AgNPs and total solution volume was maintained. Diagrams of the optical path and sample position are included in Figure S2, Supporting Information. After 5 min of irradiation, samples prepared with 1.00 to 2.00 mL of K/Au had temperature increases between 16.0 and 18.9 °C. Continued irradiation for a further 5 min increased the bulk solution temperature to between 20.9 and 24.6 °C. After ≈20 min, many of the samples reached a point where the heat gain and heat loss began to balance, and eventually, the bulk temperature decreased even though the heating laser was continuously irradiating the sample. It should be noted that the samples with the broadest properties in the NIR (3.00 and 4.00 mL of K/Au) did not have the drop in temperature that was observed with most of the other samples.

### 2.2 Characterization of Citrate‐Stabilized, Silver‐Template Hollow Gold Nanoshells After Photothermal Irradiation

**Figure 2:**
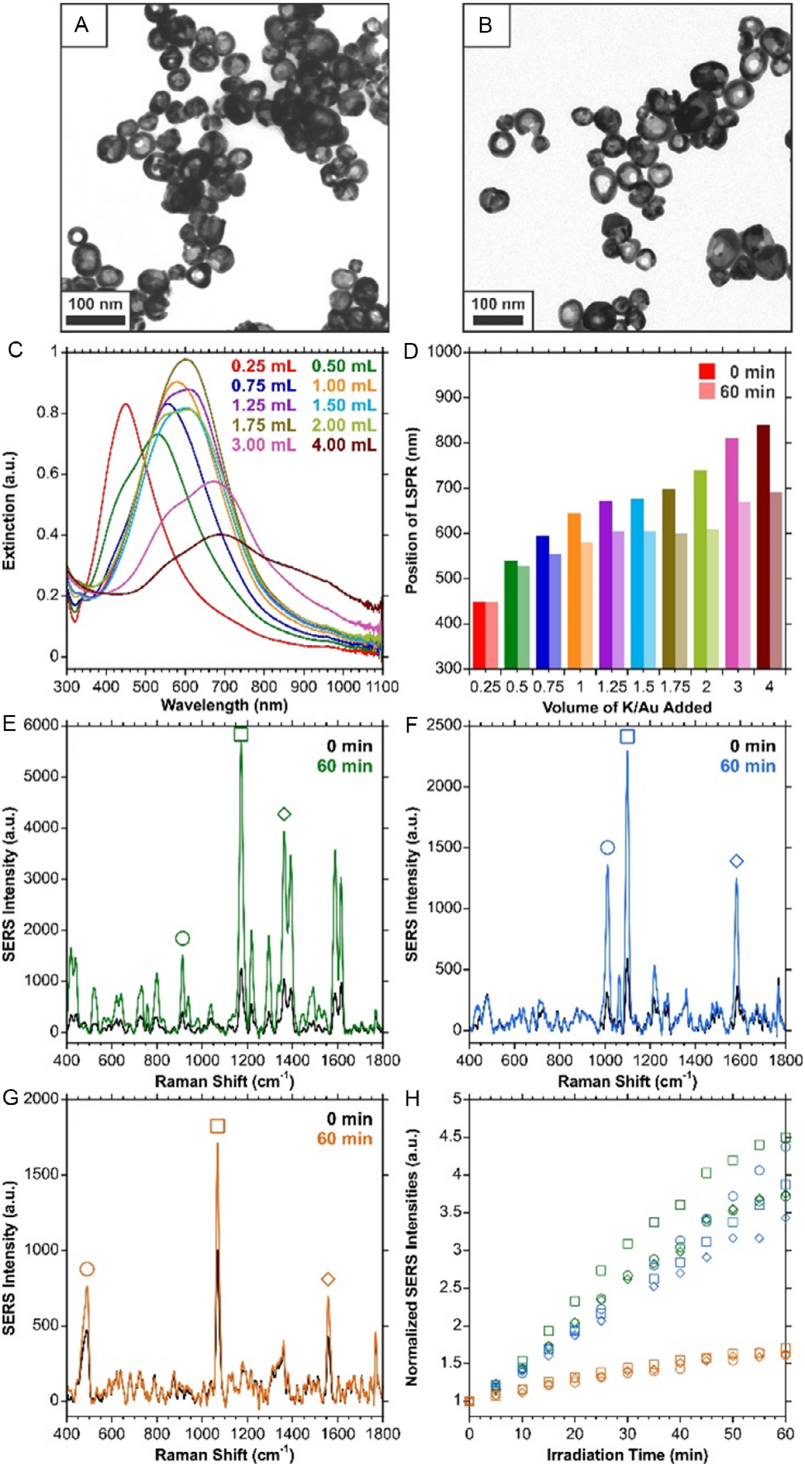
A) and B) TEM images of irradiated HGNs prepared with a 3:6:1 ratio of AgNPs: ddH_2_O: K/Au, equivalent to adding 1.00 mL of K/Au per 3.00 mL of AgNPs. C) Extinction spectra of irradiated HGNs prepared with different volumes of K/Au. D) Comparison of the LSPR shift before and after irradiation for 60 min (785 nm, 280 mW). SERS spectra before and after 60 min of irradiation for HGNs functionalized with E) malachite green isothiocyanate, F) 4‐mercaptopyridine, and G) 4‐bromothiophenol. H) Change in the normalized SERS intensity for the peaks indicated in E–G over 60 min of photothermal irradiation. SERS spectra were acquired under 785 nm (50 mW) excitation, 1 s acquisition, are an average of 10 accumulations, and baseline corrected.

After photothermally irradiating the samples, typically for 45 or 60 min, the solution of HGNs noticeably changed from blue to purple. The samples were subsequently characterized to understand the changes that occurred. Owing to the heterogeneity of the HGNs, it was difficult to see if there were significant morphological changes (**Figure** **2**A,B). Instead, extinction spectroscopy and SERS measurements were carried out.

A comparison of the extinction spectra after irradiation (Figure 2C,D, Figure S3, Table S2, Supporting Information), shows that there is a distinct blueshift in the LSPR. Importantly, this phenomenon of the LSPR blueshifting was consistently observed across different batches of HGNs prepared by different researchers and at different times (Figure S4, Table S3, Supporting Information). This change in extinction, particularly at the 785 nm excitation wavelength used for photothermal irradiation (Table S2, Supporting Information) can be used to explain the decrease in temperature during continual irradiation for some of the samples. As the optical properties shifted, the extinction at 785 nm also changed. Thus, the heating capabilities under this wavelength of irradiation changed. In the case of the 3.00‐ and 4.00 mL K/Au samples, although the optical properties shifted, the change in extinction at 785 nm was less than the sample prepared with 2.00 mL of K/Au (0.087 and 0.014 a.u., vs. 0.275 a.u., respectively). As such, it is unsurprising that the temperature did not decrease with continual irradiation. In addition to the blueshift, a new peak or shoulder was introduced at a shorter wavelength compared to the main LSPR. This could indicate a change in morphology in the structure, such as a collapse of the hollow interior, or a change in the number of holes in the shell,^[^ ^34^ ^]^ resulting in different populations of the structures compared to the original sample. Overall, for AgHGNs with LSPRS between 620 and 720 nm (Figure S4, Supporting Information), an average blueshift of 70 ± 20 nm (0.20 ± 0.06 eV) was observed (Table S3, Supporting Information).

Previous studies involving HGNs derived from AgNPs have observed that the optical properties can change under various conditions. Goodman et al. noted that their HGNs exhibited poor stability under photothermal irradiation,^[^ ^34^ ^]^ and would undergo a size‐dependent blueshift. By TEM, the authors observed that the HGNs appeared to leach Ag into the solution. It should be noted that the authors also sonicated their HGNs as a part of the synthesis. Doing so may further introduce defects into the shell or make the HGNs susceptible to morphological changes. It was determined that this could be prevented by coating the HGNs in thiolated polyethylene glycol (SH‐PEG). That work also noted that, without the SH‐PEG coating, the HGNs were pH unstable. A similar observation regarding pH‐induced changes was also observed by Zhang et al. where the authors reported a pH‐induced reduction of silver within the core of the HGNs.^[^ ^35^ ^]^ The silver‐filled nanostructures were subsequently applied to LSPR‐based sensing.^[^ ^36^ ^]^ Although these studies noticed structural changes in the HGNs, both groups only considered the change in the LSPR and never explored any other optical properties. It is also worth recognizing that a change in the optical properties under photothermal irradiation is not always observed for HGNs.^[^ ^27^ ^]^ It is therefore important to further understand why the optical properties of the HGNs prepared in this work do undergo these dramatic changes.

Beyond the blueshift in the LSPR, we observed that if the sample was functionalized with a Raman reporter, the SERS signal would increase several‐fold when irradiated photothermally. SERS spectra were collected every 5 min during photothermal irradiation for samples functionalized with either malachite green isothiocyanate (MGITC), 4‐mercaptopyridine (4‐MPy), or 4‐bromothiophenol (4‐BrTP). A comparison of the spectra before and after photothermal irradiation is shown in Figure 2E–G, with Figure 2H showing the progression of the normalized SERS intensity over time. Of these three compounds, MGITC and 4‐MPY exhibited a several‐fold increase in the SERS intensities, while 4‐BrTP increased by about 40%. MGITC was used for all subsequent SERS studies. To ensure that the SERS increase was not the result of the solution temperature being above room temperature and that the change was permanent, an MGITC functionalized sample was photothermally irradiated and allowed to cool to room temperature for four hours (Figure S5, Supporting Information). As expected, cooling the sample back to room temperature did not change the SERS signal, indicating that the new SERS response is a permanent change. Furthermore, as with the extinction spectra, Figure S6, Supporting Information, compiles the changes in SERS signals across a series of measurements by different researchers. The relative increases in the SERS responses do vary (Figure S7, Supporting Information), but importantly, the phenomenon was consistently observed. There was an average fold increase of 5 ± 2 in the SERS signal for the dominant 1174 cm^−1^ peak across those measurements. For the same batch of AgHGNs irradiated on the same day, the variation in the relative SERS intensity is small (Figure S8A, Supporting Information), but when multiple batches are compared (Figure S8B‐K, Supporting Information), the large variation is once again observed (Figure S8L, Supporting Information), with an average fold increase of 8 ± 2 for the 1174 cm^−1^ peak after 60 min of photothermal irradiation.

### 2.3 The Importance of Having Citrate Present

**Figure 3:**
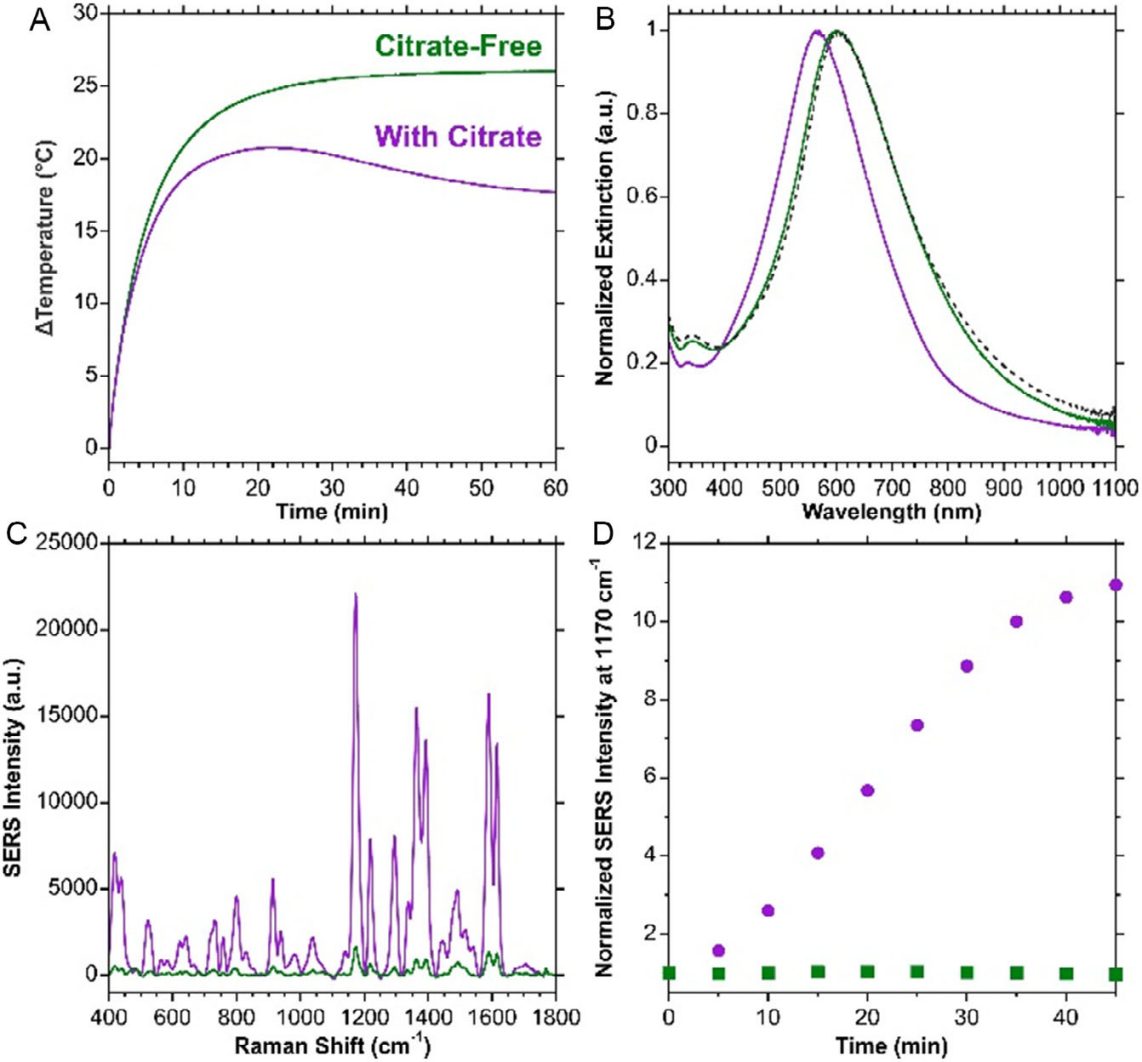
Comparison of HGNs that were prepared without adding citrate after the synthesis with those that had citrate added. A) Bulk temperature profiles for photothermal heating for 60 min under 785 nm (280 mW) irradiation, with B) the corresponding extinction from before heating as the dashed spectrum and after heating the solid lines. C) SERS spectra from different batches of HGNs as (A) and (B) functionalized with MGITC after having been photothermally irradiated for 45 min. D) Temporal analysis of the SERS intensity of the 1170 cm^−1^ vibrational mode of MGITC normalized to the intensity prior to irradiation. SERS spectra were acquired under 785 nm (50 mW) excitation, 1 s acquisition, are an average of 10 accumulations, and baseline corrected.

Citrate was added to improve the long‐term stability of the HGNs after they were synthesized, however, it is not strictly required. Therefore, citrate‐free samples were also prepared to investigate the effect that citrate has on the resulting optical properties upon photothermal heating. Interestingly, it was found that without citrate, although the heating was comparable at the beginning, the final temperatures reached after one hour were different (**Figure** **3**A). After 60 min of photothermal irradiation, the extinction spectra only changed for the sample with citrate present (Figure 3B). This further supports the results from Figure 1D, demonstrating that the decrease in bulk solution temperature over time is likely the result of the optical properties of the structures blueshifting away from the excitation wavelength, as no decrease in temperature was observed for the citrate‐free samples.

Furthermore, a comparison between the SERS spectra of the samples (Figure 3C,D) shows that in the absence of citrate, the SERS intensity does not vary with photothermal irradiation. Importantly, the trends of no blueshift or increase in SERS response were consistently observed (Figure S9, Supporting Information). It was also found that the concentration of citrate was critical, as the changes were less noticeable below a citrate concentration of 1 mM (Figure S10, Supporting Information). Therefore, the concentration range of citrate typically used to aid the stability of nanoparticles was within the concentration range that also causes these observed effects.

To understand if this was a citrate specific phenomenon, a series of experiments were performed whereby different molecules were added to citrate‐free HGNs. In the synthetic protocol used by Zhang et al. to prepare Ag nanoplates,^[^ ^37^ ^]^ NaBH_4_ acted as the reducing agent, and H_2_O_2_ was an oxidative etchant that aided in the formation of planar twinned seeds. It was known that citrate interacts with the Ag (111) facets,^[^ ^38^, ^39^ ^]^ but the authors also observed that other molecules containing at least two carboxyl groups separated by two carbon atoms could help stabilize the nanoplates,^[^ ^37^ ^]^ thus producing silver nanoplates in high yields. We therefore took inspiration from that work and investigated how isocitrate, succinate, malonate, and *cis*‐aconitate compared to citrate in changing the optical properties of the HGNs (Figure S11, Supporting Information).

These results indicate that citrate plays a critical role in the observed changes in the optical properties of the HGNs, as none of the other molecules yielded the same dramatic changes as citrate. The optical properties did slightly change when isocitrate was used, but the changes were smaller. Interestingly, during an investigation into the mechanisms behind the photomediated synthesis of Ag nanoplates, it was found that isocitrate could produce nanoprisms and other irregular structures in low yield.^[^ ^40^ ^]^ The results of Figure 2 and 3, and S11, Supporting Information, therefore support the notion that the at the very least, the blueshift in the optical properties could be the result of a chemical reaction involving the citrate used to help stabilize the HGNs.

### 2.4 The Importance of Temperature and Photothermal Irradiation

**Figure 4:**
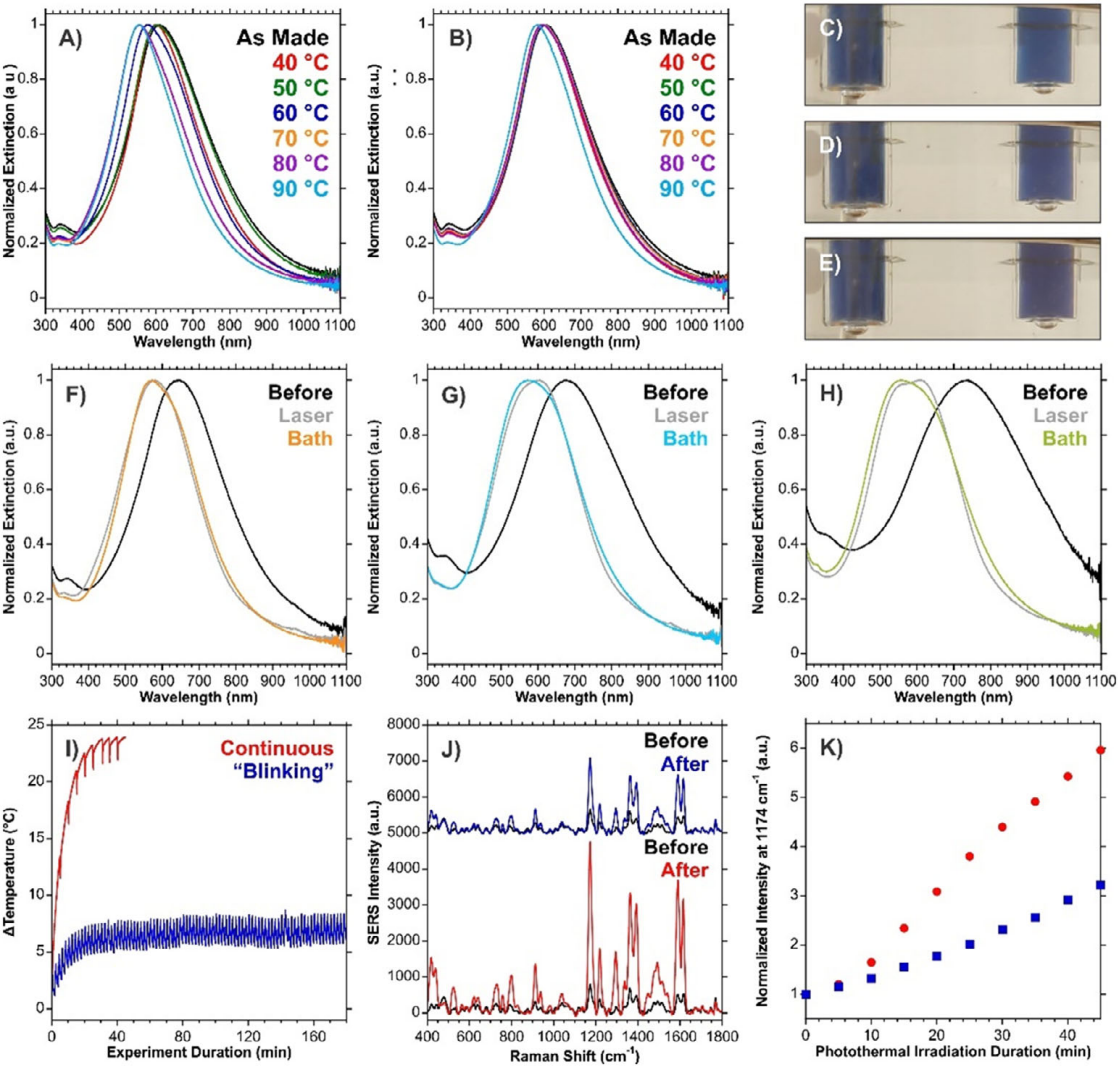
Comparison of normalized extinction spectra of A) citrate‐stabilized and B) citrate‐free HGNs heated with an oil bath at defined temperatures for 20 min. Each spectrum is the average of 3. Images of vials containing citrate‐free (left), and citrate‐stabilized (right) HGNs taken after C) 0 min, D) 5 min, and E) 10 min of heating in an oil bath at 70 °C. Comparison of the normalized extinction spectra of citrate‐stabilized HGNs before and after photothermal heating and heating at 70 °C in the oil bath for 20 min for HGNs prepared with volume ratios of F) 3:6:1, G) 3:5.5:1.5, and H) 3:5:2 for AgNPs: ddH_2_O: K/Au. I) Temperature heating profiles for citrate‐stabilized HGNs that underwent 45 min of continuous 785 nm (280 mW) irradiation or “blinking” whereby the shutter was opened and then closed repeatedly for 30 and 90 s durations respectively. J) Changes in the SERS intensities for MGITC functionalized HGNs after continuous or “blinking” exposure. K) Variation in the intensity of the 1174 cm^−1^ vibrational mode of MGITC over the duration of photothermal irradiation normalized to the intensity prior to irradiation. SERS spectra were acquired under 785 nm (50 mW) excitation, 1 s acquisition, are an average of 10 accumulations, and baseline corrected. The spectra in (J) were offset for clarity.

By its very nature, photothermal irradiation combines both light and temperature as reaction conditions at the surface of nanomaterials. To better understand these effects, we sought to explore means of separating out these factors. Rather than photothermally irradiating the samples, glass vials with 1 mL of citrate‐free and citrate‐stabilized HGNs were left in an oil bath at different temperatures for 20 min. As the extinction spectra of **Figure** **4**A,B show, there is once again a distinct difference between the behavior of citrate‐stabilized and citrate‐free HGNs. For the citrate‐stabilized HGNs, temperatures below 50 °C, the typical threshold during photothermal irradiation, were insufficient to cause a shift in the LSPR (Figure 4A). When the temperature was raised to 60 °C, a marginal blueshift was observed, which further shifted upon increasing the temperature. For citrate‐free samples (Figure 4B), a temperature of 90 °C was needed for a blueshift of 20 nm (0.07 eV) to occur. The results are summarized in Table S4, Supporting Information. Optical images taken during the heating at 70 °C demonstrate the characteristic change from blue to purple for the HGNs for the citrate‐stabilized samples, but not the citrate‐free (Figures 4C–E).

HGNs prepared with different ratios of galvanic replacement were heated in an oil bath at 70 °C for 20 min and evaluated with respect to photothermal irradiation (Figure 4F–H, and Figure S12, Supporting Information). For the samples prepared with the least amount of galvanic replacement, there was minimal discrepancy between laser and bath heating. As the amount of galvanic replacement increased, a greater shift occurred with oil bath heating. It is possible that the increase in galvanic replacement leads to less stable HGNs, which would therefore be more susceptible to changes when the higher temperatures were used during the oil bath heating compared to the photothermal heating. Overall, the increase in temperature may be playing a factor, but it is not known how any differences between bulk, surface, and interior temperatures would affect this process. This is especially the case since, when the temperatures achieved by photothermal and block heating were comparable (Figure S13, Supporting Information), only the photothermally irradiated HGNs underwent the blueshift and increase in SERS response. Under photothermal irradiation, the temperature at the surface of the nanoparticle is likely to be greater than that of the bulk. As such, under photothermal irradiation, the surface temperature may reach the necessary threshold temperature, whereas the bulk heating does not.

When collecting the SERS spectra during photothermal irradiation, it was necessary to temporarily block the photothermal beam path to prevent interference during the SERS measurements. This resulted in noticeable and rapid decreases in the temperature of the bulk solution. Upon the beam irradiating the sample, the temperature would increase (red curve of Figure 4I). It was therefore postulated that this could be exploited as a means of maintaining a lower solution temperature while keeping the irradiance of the photothermal laser on the HGNs fixed. As opposed to continually irradiating the sample, a shutter was opened for 30 s and then closed for 90 s. This is herein referred to as “blinking”. By performing this process over the course of three hours, the total illumination time between the samples remained the same (45 min). The heating curves (Figure 4I) indicate that this approach yielded the desired effect of having different bulk temperatures. A comparison of the SERS spectra before and after the total experiment shows that although the SERS intensities before irradiation were comparable, the continuously irradiated sample showed a higher final SERS intensity (Figure 4J,K), however, neither sample reached a plateau where the SERS signal no longer increased. Given that both samples experienced the same duration of exposure to the 785 nm heating beam, it is unlikely that the observed effects are solely a light‐driven process, as, if they were, the changes should be the same. Instead, a combination of temperature and light exposure is critical for the observed optical changes to occur at a bulk temperature less than 50 °C. Upon photothermal irradiation, a rapid temperature increase occurs at the surface of the HGNs, and is quickly transferred to the aqueous environment at the pico‐ to nanosecond timescale.^[^ ^41^ ^]^ In the case of the HGNs, there is both the inner and outer nanoshell environments that need to be considered. Given that the heat within the inner core would also need to transfer to the bulk exterior solution, it is likely that the hollow interior maintains a higher temperature longer. These processes should be independent of whether or not the sample is continuously irradiated. However, the rate of heat transfer is likely to be affected by the difference between the bulk and surface temperatures. When the difference between bulk and surface temperatures is less, then the rate of heat flow would be slower, resulting in a higher surface temperature for longer. In the nanoscale hollow interior, the heat transfer involves both the metal but also into the bulk through the porous shell. This discrepancy between surface and bulk temperatures may explain why, in the oil bath, higher bulk temperatures are required to achieve the shift in the optical properties, as it is not the bulk temperature that is the most important aspect, but the temperature at or near the surface of the HGNs.

### 2.5 The Importance of Silver Being Present

As was demonstrated in Figure 1C, the addition of higher volumes of K/Au allows for continued galvanic replacement. Therefore, at the 3:6:1 ratio (volumes of AgNPs: ddH_2_O: K/Au) used throughout this work, it is unlikely that any excess AuCl_4_^−^ remains for the proposed reduction reaction to involve gold. Based on literature on HGNs and the results obtained thus far, it was proposed that silver (Ag^+^ or Ag^0^) must be a factor. Origins of this silver include: Ag^+^ left in the solution from the galvanic replacement, the oxidative dissolution of remaining Ag^0^ in the presence of O_2_,^[^ ^42^, ^43^ ^]^ as well as rearrangement of Ag^0^ present within the shell.

Two methods were considered to determine the importance of silver: repeated cycles of centrifugation and the use of an alternative sacrificial template. First, samples of AgHGNs underwent repeated rounds of centrifugation followed by resuspension in 3 mM citrate or ddH_2_O and were then photothermally irradiated (Figure S14, Supporting Information). With successive cycles of centrifugation and resuspension, the degree of the blueshift became less after photothermal irradiation. As centrifugation should remove free Ag^+^ ions, the minimal shift after three rounds of centrifugation supports our belief that the Ag^+^ ions are playing an important role in the changes to the optical properties. The more effective way of evaluating the importance of silver was to prepare HGNs using CoNPs as the sacrificial template (HGN_Co_s). Under these circumstances, no source of silver was present. Given that this work has established that citrate plays a role in the observed phenomena, it is important to recognize that citrate is used during the synthesis of the HGN_Co_s and that the HGN_Co_s were stabilized in a 2 mM solution of citrate.

**Figure 5:**
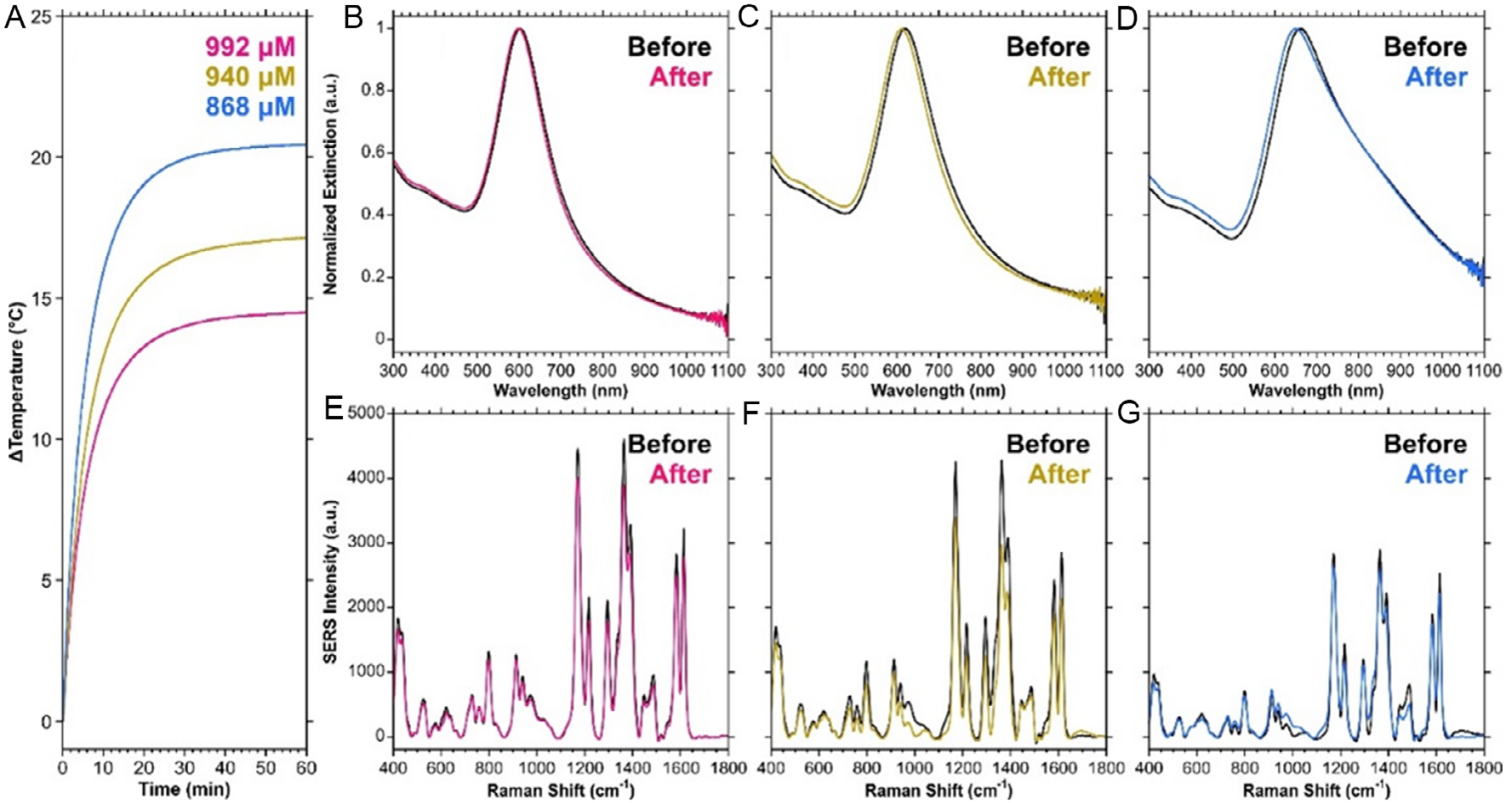
Photothermal, optical, and SERS properties of HGN_Co_s prepared with different concentrations of HAuCl_4_. A) Temperature curves for samples at an optical density of ≈1 and photothermally irradiated for 1 h. Normalized extinction spectra before and after one hour of photothermal irradiation (785 nm, 280 mW) for samples prepared with 50 mL of B) 992, C) 940, and D) 868 μM HAuCl_4_. SERS spectra of HGN_Co_s functionalized with MGITC before and after one hour of photothermal irradiation for samples prepared with 50 mL of E) 992, F) 940, and G) 868 μM HAuCl_4_·3H_2_O. SERS spectra were acquired under 785 nm (50 mW) excitation, 1 s acquisition, are an average of 10 accumulations, and baseline corrected.

The photothermal heating curves for HGN_Co_s with LSPRs between 600 and 660 nm, taken with the HGN_Co_s at an optical density of ≈1, are shown in **Figure** **5**A. Unlike the HGNs derived from AgNPs, the temperature increased plateaued after one hour of photothermal irradiation as opposed to an increase followed by a gradual decrease (Figure 1D). Furthermore, the extinction spectra remain similar after irradiation (Figure 5B–D) while the SERS signal decreases (Figure 5E–G). These results demonstrate that the choice of sacrificial core is important in the observed optical changes upon irradiation. In the absence of silver from the galvanic replacement of the AgNPs with K/Au, the blueshift and increase in the SERS intensity does not occur.

### 2.6 Proposed Mechanisms for the Anomalous Changes

Individually, citrate, temperature, and silver do not cause the observed changes. Even combining two of the three does not appear to yield the blueshift in the LSPR and unprecedented increases in the SERS intensity. Instead, the three have a synergistic relationship that causes the changes in the optical properties upon photothermal irradiation of the HGNs.

**Figure 6:**
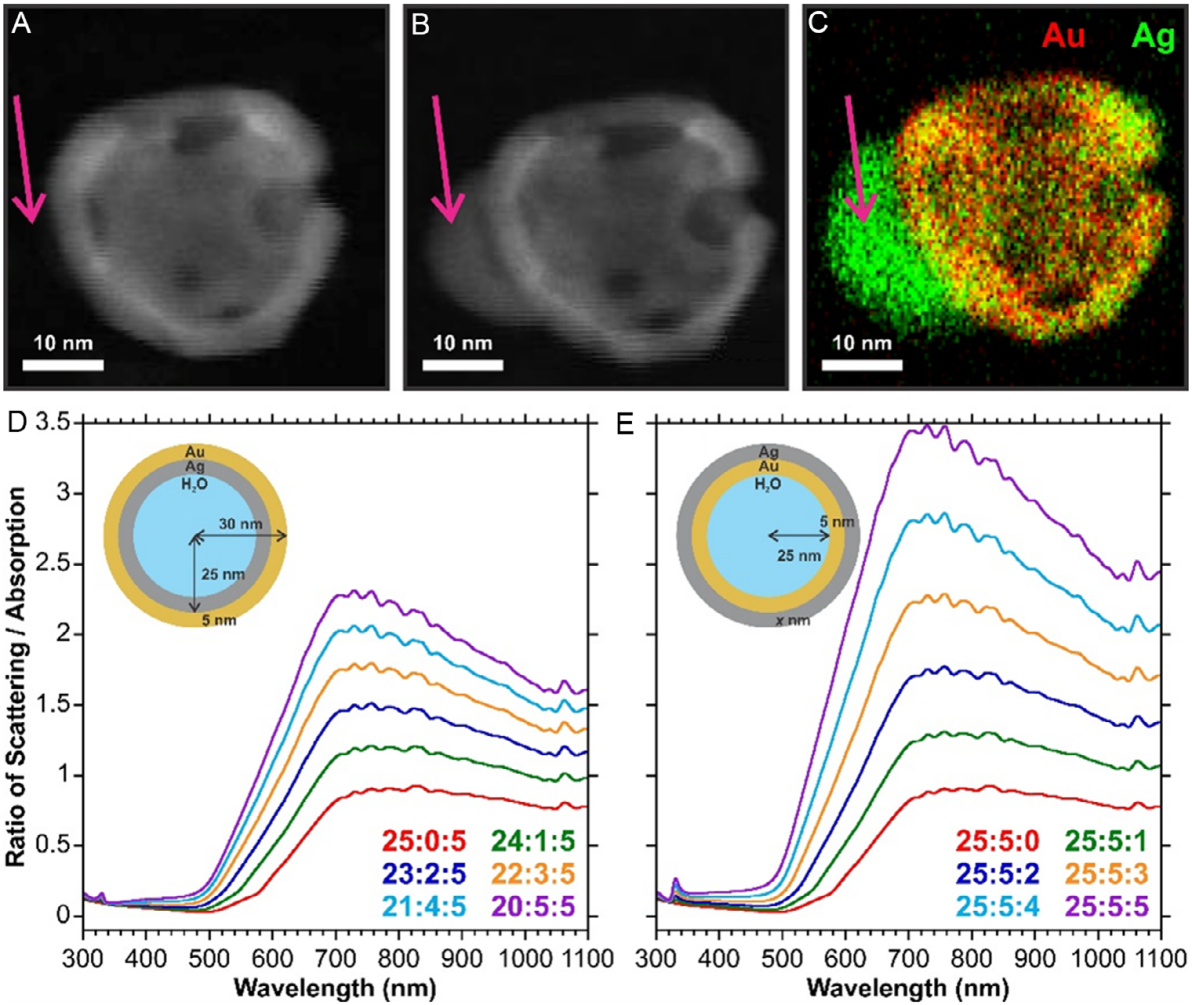
HAADF images of an HGN taken under constant exposure A) initial and B) 50 s later. C) EDX image taken at the end of the measurement. The purple arrows indicate a location where a silver deposit formed during the measurement. Ratios of the calculated scattering cross‐section to absorption cross‐section for the different HGNs using a Mie theory calculator for the formation of a D) interior and E) exterior silver shell.

Although none of the TEM images were sufficient to show the type of dramatic changes demonstrated by others, it is necessary to remember that the HGNs prepared are extremely heterogeneous. Relying simply on a few TEM images is not sufficient to fully understand what is happening across all the different geometries present. High‐angle annular dark field scanning TEM (HAADF‐STEM) imaging combined with energy dispersive X‐ray (EDX) spectroscopy was carried out to further understand the HGN composition and structure (Figures S15–17, Supporting Information). When the samples were being imaged, morphological changes were observed (Figure S15, Supporting Information), but more importantly, a silver deposit formed at the surface (**Figure** **6**A–C, Figures S16 and 17, Supporting Information). It was previously demonstrated that the presence of Ag^+^ ions from within the core of hollow AgAu nanorods could be used to reshape the interior using the electron beam of a scanning tunneling electron microscope.^[^ ^44^ ^]^ In another work,^[^ ^45^ ^]^ it was observed that an electron beam could be used to help grow silver nanoplates in the presence of AgNO_3_ and silver nanocrystals in close proximity. The purple arrows of Figure 6A–C indicate that the growth of the Ag deposit appears to occur at an existing hole within the shell. The conditions used during the HAADF‐STEM measurements, such as the presence of an electron beam, are certainly not the same as during photothermal measurements. However, the observed morphological changes that occur during the HAADF‐STEM measurements may represent extreme versions of what could occur during photothermal irradiation. As such, inferring from these observations, we propose the following mechanisms for the observed anomalous changes.

The galvanic replacement of the AgNPs to form the HGNs results in the generation of Ag^+^. These Ag^+^ ions are free to diffuse throughout the solution and may accumulate within the interior of the HGN through diffusion via the porous shell.^[^ ^21^, ^29^, ^46^, ^47^, ^48^ ^]^ Furthermore, the surfaces of the HGNs are rough, likely supporting a variety of crystal phases. In the presence of oxygen and light, oxidative dissolution of Ag^0^ to Ag^+^ is known to occur.^[^ ^49^ ^]^ This process is size dependent, with smaller structures undergoing this process faster. It has also been reported that this process is temperature dependent,^[^ ^50^ ^]^ with an increase in temperature resulting in faster dissolution. It was also previously reported that the oxidative dissolution of Ag^0^ is slower for citrate‐stabilized nanoparticles compared to other capping ligands.^[^ ^50^, ^51^, ^52^ ^]^ Even with this knowledge, we propose that both sources of Ag^+^, residual from galvanic replacement and oxidative dissolution, are likely to occur with the HGNs.

In the presence of a high concentration of AgNO_3_ (150 mM), it is possible for Ag^0^ to form at the surface of gold nanorods under white light illumination in the absence of a reducing agent.^[^ ^53^ ^]^ Critically, in our work, in the absence of citrate, this did not occur. Furthermore, the theoretical maximum silver content is ≈0.3 mM (calculation in the Supporting Information), far lower than the 150 mM concentration used in that other work. The combination of these two factors leads us to believe that the mechanism at play is dependent on the presence of the citrate capping/stabilizing ligand. The light sensitivity of the combination of silver and citrate is well‐established.^[^ ^54^ ^]^ Drawing parallels to other structures and reactions,^[^ ^55^, ^56^, ^57^, ^58^ ^]^ we surmise that under photothermal irradiation, the light induced reduction of Ag^+^ to Ag^0^ occurs, resulting in the formation of nanoscale Ag^0^ deposits at the surface of the HGNs. This process likely benefits from having citrate at the surface of the structure, through its coordination with the silver atoms,^[^ ^59^, ^60^ ^]^ which in effect captures the Ag^+^ and keeps it near the surface,^[^ ^43^ ^]^ as well as having citrate in solution to replenish the citrate used in the reaction. Parallels can therefore start to be drawn to plasmon‐driven transformations involving AgNPs,^[^ ^38^ ^]^ specifically the plasmon‐driven synthesis of Ag shells around Au nanoplates.^[^ ^61^ ^]^ In that work, AgNPs were the source of Ag^+^ and Ag^0^, with the shell growth being dependent on exciting the optical properties of the Au nanoplate using a 1064 nm light source. When a visible excitation was used, only transformations of the AgNPs to Ag nanoplates were observed, with no shell growth on the Au nanoplates.

It is also necessary to recognize that the classical synthesis of citrate‐stabilized AgNPs is temperature dependent, with boiling being a common reaction condition.^[^ ^62^ ^]^ However, it has been demonstrated that the temperature does not need to be boiling for the growth of AgNPs to occur when citrate is present.^[^ ^63^, ^64^ ^]^ The work of Patra et al. discussed the importance of nanoconfinement in the decomposition of silver citrate complexes to form AgNPs.^[^ ^65^ ^]^ Under normal conditions, when the ratio of [citrate]/[Ag^+^] is sufficiently high (>>1), the silver citrate complexes are thermally stable at 75 °C. However, when nanoscale confinement is introduced, the reaction can proceed, and AgNPs form. With a citrate concentration of 3.3 mM and a maximum Ag^+^ content of 0.3 mM, a minimum ratio of 11 occurs. This is likely within the regime whereby nanoscale confinement, such as the hollow interior or porous shell, provides opportunities for the decomposition of the silver citrate to occur. Though the bulk temperatures achieved during the photothermal irradiation of the HGNs are far from boiling, the temperature at the surface of the HGNs and within the hollow aqueous interior will be greater than the bulk temperature,^[^ ^66^ ^]^ and could reach a necessary threshold for temperature‐based reduction to also occur. Eventually, this process of growth and/or rearrangement of silver atoms reaches a conclusion as the Ag^0^ most susceptible to oxidative dissolution and any remaining Ag^+^ ions are consumed. It is therefore believed that a combination of factors is contributing as opposed to just one. A mechanism that assumes purely plasmonic driven processes, whereby hot charge carriers are generated by exciting the optical properties of the HGNs, ignores the observed temperature increase. Likewise, the temperature increase alone is insufficient, as in the bulk measurements, higher temperatures were required to drive the change in the optical properties than what were found during photothermal irradiation.

Understanding the blueshift in the optical properties can be explained by combining inferences from the HAADF‐STEM images and previous literature. The optical properties of gold nanoshells were modeled in the early work on the nanostucture,^[^ ^12^ ^]^ later serving as the base structure for the plasmon hybridization model.^[^ ^67^ ^]^ In short, for a fixed total diameter, as the shell thickness grows, the hollow interior decreases, and a blueshift in the LSPR is observed. Using a Mie theory calculator,^[^ ^68^ ^]^ a comparison between the extinction, scattering, and absorption coefficients was made for different geometries: i) a filling of the interior with a thin silver shell (Figure S18A, Supporting Information) and ii) deposition of a thin silver shell onto the exterior (Figure S18B, Supporting Information). In these calculations, the refractive index of the hollow interior and surrounding media was set to 1.33, with the optical constants of silver and gold derived McPeak et al.^[^ ^69^ ^]^ In both instances, there is a blueshift in the LSPR. These calculations assume a uniform growth of the silver layer. Figure 6C shows that the growth can be anisotropic. This anisotropic growth could explain the second peak or shoulder commonly observed in the extinction spectra of the photothermally irradiated HGNs. If the behavior is closer to that of the silver coating of Au nanoplates,^[^ ^61^ ^]^ then a more uniform thin silver shell would form. Alternatively, if the hollow interior of the HGN collapses and the Au content is kept constant (Figure S19, Supporting Information), the shell thickness increases until such time that the HGN becomes an AuNP. In both scenarios, a change in shell thickness, and thus optical properties, would occur.

The origin of the increased SERS signal is more challenging, especially as the optical properties shift away from the 785 nm excitation used for the SERS measurements. The theoretical changes in the scattering to absorption ratios may provide an explanation (Figure 6D,E). In both cases, the change in the core‐to‐shell ratio alters the scattering‐to‐absorption ratio. At the 785 nm excitation wavelength used, the scattering contribution in the extinction spectrum increases relative to absorption. A major contribution to the SERS response is the ability of the metallic nanoparticle to scatter light.^[^ ^70^ ^]^ Increasing the scattering to absorption ratio would therefore favor SERS over heat generation for a given excitation wavelength.^[^ ^71^ ^]^ As such, we believe that the cause in the blueshift in the LSPR, the change in the core‐to‐shell ratio, is simultaneously, also the cause of the increase in the SERS signal.

## 3 Conclusion

We have consistently observed a series of anomalous and counterintuitive optical effects involving photothermally irradiated HGNs. The peculiar changes in the optical properties of photothermally irradiated HGNs require the presence of citrate, heat, and silver from the AgNP templates. Without all three factors, the changes do not occur. The blueshift in the LSPR can be ascribed to the formation of silver deposits at the surface of the HGNs. The formation of these deposits requires the presence of Ag^+^, which forms during the galvanic replacement of the AgNPs with AuCl_4_^−^ and citrate. The citrate added to stabilize the HGNs can also capture the Ag^+^ and form an insoluble silver citrate complex at the surface of the HGNs. Upon photothermal irradiation, the Ag^+^ is reduced to Ag^0^, resulting in a change in the core‐to‐shell thickness. As such, a blueshift in the LSPR occurs. We surmise that this change in the optical properties also gives rise to the counterintuitive increase in the SERS scattering. When the core‐to‐shell ratio changes, the scattering contribution in the extinction also increases. Thus, even though the LSPR shifts away from the SERS excitation, with the increase in scattering efficiency, more incident photons are scattered as opposed to absorbed. And as a result, there is an increase in the SERS response. This new insight will allow HGNs to be used to their full potential by other researchers in the field and take advantage of these unique optical properties.

## 4 Experimental Methods

4.1

4.1.1

##### Materials

Hydroxylamine hydrochloride (HONH_2_·HCl), sodium hydroxide (NaOH), silver nitrate (AgNO_3_), chloroauric acid trihydrate (HAuCl_4_·3H_2_O), trisodium citrate dihydrate (citrate, Na_3_C_6_H_5_O_7_·2H_2_O), cobalt chloride hexahydrate (CoCl_2_·6H_2_O), sodium borohydride (NaBH_4_), 4‐MPY, and 4‐BrTP were all purchased from Sigma Aldrich. Potassium carbonate (K_2_CO_3_) was acquired from VWR. MGITC was purchased from Thermo Fisher Scientific.

##### Synthesis of Silver Nanoparticles and Hollow Metallic Nanoshells Derived from the Galvanic Replacement of Silver

Prior to the synthesis of the nanoparticles, all glassware was thoroughly cleaned with aqua regia. *CAUTION: aqua regia is highly corrosive and must be handled carefully*. Glassware was thoroughly rinsed with water, including doubly distilled water (ddH_2_O). Several NaOH pellets were added to the flask and dissolved in ddH_2_O, and left to further neutralize any remaining acidity, and then re‐rinsed with ddH_2_O. The synthesis of AgNPs was adapted from a previously described method.^[^ ^34^ ^]^ Briefly, 15.7 mg of NaOH was dissolved in 120 mL of ddH_2_O in a 250 mL Erlenmeyer flask. Separately, 14 mg of HONH_2_·HCl was dissolved in 1.33 mL of ddH_2_O, and 22.3 mg of AgNO_3_ dissolved in 13.3 mL of ddH_2_O. The HONH_2_·HCl solution was added to the NaOH solution and allowed to mix under rapid stirring for 5 min at room temperature, after which the silver nitrate solution was rapidly added, and the stirring continued for at least 15 min at room temperature. The final solution of AgNPs had a yellowish‐brown color in the flask, though it appeared more yellow once stored in a cleaned Duran. In a typical synthesis where a new batch of AgNPs was being made, the stirring was maintained until the AgNPs were needed for the preparation of the hollow metallic nanoshells. Once the AgNPs were prepared, a solution of K/Au was made. Here, 10 mg of HAuCl_4_·3H_2_O was dissolved in 1 mL of ddH_2_O, and subsequently added to 12.5 mg of K_2_CO_3_ in 50 mL of ddH_2_O. This solution was mixed and left undisturbed for at least 30 min prior to being used, but was used within 1 h of being made. In a typical galvanic replacement, 30 mL of the as‐prepared AgNPs were mixed with 60 mL of ddH_2_O under constant, rapid stirring in an Erlenmeyer flask. 10 mL of the aged K/Au solution was rapidly added under continuous stirring. Under these conditions, immediately upon the addition of the K/Au, the color of the solution rapidly changed from yellow to brown to purple and then to blue. The solution was further mixed for 10 min. To this solution of HGNs, 2 mL of 0.5% w/v Na_3_C_6_H_5_O_7_·2H_2_O in ddH_2_O was added. NOTE: This step was omitted for experiments involving citrate‐free samples. For experiments with different ratios of galvanic replacement, the reaction was scaled down by one order of magnitude and was reacted in clean glass vials. Therefore, 3 mL of AgNPs, 6 mL of ddH_2_O, 1 mL of the K/Au solution, and eventually 0.2 mL of the citrate solution. For said study, the ratios are described in Table S1, Supporting Information. Once prepared, samples were stored in the dark until use. The resulting HGNs exhibited a broad range of colors (Figure S11A, Supporting Information) dependent on the amount of galvanic replacement.

##### Synthesis of Cobalt Nanoparticles and Hollow Metallic Nanoshells Derived from the Galvanic Replacement of Cobalt

The CoHGNs syntheses were carried out under inert conditions by bubbling nitrogen through the solution to prevent the CoNPs from prematurely oxidizing. The method described was heavily modified from a previous protocol to better reflect current lab capabilities.^[^ ^19^ ^]^ A 250 mL three‐necked round‐bottom flask was thoroughly cleaned using a similar protocol to the one described in the previous section. In a typical synthesis, rubber stoppers were added to the two flanking necks, followed by the addition of 200 mL of ddH_2_O to the flask. An air condenser was then added to the middle neck. Nitrogen was bubbled through the solution continuously unless otherwise stated. CoCl_2_·6H_2_O (0.4 mL, 0.4 M) and Na_3_C_6_H_5_O_7_·2H_2_O (1.6 mL, 0.1 M) were injected into the solution. The solution was stirred and degassed with nitrogen for 15 min. Freshly prepared NaBH_4_ (4 mL, 0.1 M) was injected into the solution and allowed to react for a further 60 min until hydrogen evolution ceased, indicating complete hydrolysis of the reductant. At this point, the solution was a deep brown color, indicating the formation of the CoNPs. A stock 1% solution of HAuCl_4_·3H_2_O was made. 50 mL of ddH_2_O was degassed under nitrogen while the CoNPs were being synthesized. A corresponding amount of the 1% solution was added to the 50 mL of degassed ddH_2_O to prepare 50 mL of HAuCl_4_·3H_2_O with concentrations ranging from 868 to 992 μM. 33 mL of this dilute HAuCl_4_ solution was injected into the solution of CoNPs and allowed to mix under stirring for 10 min. The solution was then exposed to air by ceasing the flow of nitrogen and removing the rubber stoppers. An obvious color change from brown to purple, dark blue, or teal (depending on the concentration of Au added) was observed, indicating that the galvanic replacement had occurred. Finally, trisodium citrate (2 mL, 0.1 M) was added to stabilize the CoHGNs. Postsynthesis, the CoHGN solution was concentrated through centrifugation (6000 rpm, 3.3 rcf) and the precipitate was redispersed in trisodium citrate solution (2 mM) to give a final optical density of 1.

##### Characterization of Nanoparticles

For characterizing the HGNs before and after heating, extinction measurements were carried out using a Cary 60 UV–vis‐NIR spectrometer, scanning from 300 to 1100 nm at a scan rate of 4800 nm min^−1^. TEM imaging was carried out at the University of Glasgow using a JEOL JEM‐1400Flash, and an 80 kV accelerating voltage. Samples were prepared by drop casting 1 μL of sample, onto 200 Mesh Carbon‐coated copper TEM grids. HAADF‐STEM imaging combined with EDX measurements were performed using a Thermo Fisher Scientific Tecnai Osiris TEM operated at 200 kV (University of Antwerp).

##### Photothermal Heating

For all photothermal heating experiments, 500 μL of the colloidal solution was added to a 1.75 mL glass vial. The vial was then placed into a 3D‐printed holder. A custom‐built photothermal set‐up was used, where a collimated 785 nm laser (300 mW at the source) was directed and focused onto the sample. If needed, a welded tip fiberglass thermocouple (TCI Direct, 401‐941) was added into the solution, but positioned outside of direct contact with the beam. A TC‐08 thermocouple data logger (Pico Technology Ltd.) and Picolog software was then used to record the temperature at 1 s intervals. The shutter associated with the heating beam was briefly closed when the SERS spectra were collected, and then reopened once the spectra were acquired. A representative diagram of the optical paths is shown in Figure S2, Supporting Information.

##### Surface‐Enhanced Raman Scattering Measurements

Prior to SERS measurements, HGNs were functionalized with a Raman reporter. In an Eppendorf tube, different volumes of 10 μM MGITC in water was added to 1 mL of the prepared HGNs to obtain a sufficient SERS response that could be observed, whilst minimizing aggregation of the HGNs. Typically, volumes between 7.5 and 15 μL were sufficient. Samples were placed onto a roller and allowed to mix for at least 60 min prior to being centrifuged (3000–4000 rpm (0.8–1.5 rcf) for 20 min). A similar approach was used for the experiments involving the other Raman reporters. For the HGN_Co_s, a higher concentration of MGITC was needed (15 μL of 100 μM MGITC per 1 mL of CoHGNs). Once again, a similar approach was used to evaluate different MGITC concentrations to minimize aggregation. A Wasatch Photonics 785 nm Raman probe was used to irradiate the HGNs and collect the backscattered light. Prior to acquiring the SERS spectra, the shutter of the photothermal laser was closed so that the sample was no longer being photothermally irradiated. The SERS spectra were then collected using a Wasatch Photonics 785 nm spectrometer. A laser power of 50 mW with an integration time of 1 s was used, and 10 spectra were collected. After the spectra were acquired, the shutter was opened, and photothermal irradiation continued. SERS spectra were processed using a custom Python 3 script, which determined a 3rd order polynomial baseline for each spectrum and subsequently subtracted it from the original spectrum to yield the baseline correction. The 10 spectra were then averaged.

## Supporting Information

Supporting Information is available from the Wiley Online Library or from the author.

## Conflict of Interest

The authors declare no conflict of interest.

## Supporting information

Supplementary Material

## Data Availability

The data will be available at https://doi.org/10.15129/634c51bf‐5283‐4a5f‐8371‐f9ed72f187cb.
