# Supplementary material for: Unveiling the Unprecedented Optical Properties of Citrate‐Stabilized Hollow AgAu Nanoshells Under Photothermal Irradiation
Source: Small Sci. 2025 Dec 4;6(1):e202500494. doi: 10.1002/smsc.202500494 (PMC12794676; doi:10.1002/smsc.202500494)
Supplement: Supplementary file 1 — Supplementary Material [file SMSC-6-e202500494-s001.pdf]

Supporting Information

Unveiling the Unprecedented Optical Properties of Citrate-Stabilized Hollow AgAu Nanoshells Under Photothermal Irradiation

*Gregory Q. Wallace, Jennifer Gracie, Amritpal Singh, Benjamin Clark, Kellie Jenkinson, Sara Bals, W. Ewen Smith, Tell Tuttle, Karen Faulds, and Duncan Graham**

Table S1. Volume ratios used in the preparation of HGNs with varying amounts of galvanic replacement.

Sample	Volume of AgNPs	Volume of ddH_2O	Volume of K/Au
1	3 mL	6.75 mL	0.25 mL
2	3 mL	6.5 mL	0.5 mL
3	3 mL	6.25 mL	0.75 mL
4	3 mL	6 mL	1 mL
5	3 mL	5.75 mL	1.25 mL
6	3 mL	5.5 mL	1.5 mL
7	3 mL	5.25 mL	1.75 mL
8	3 mL	5 mL	2 mL
9	3 mL	4 mL	3 mL
10	3 mL	3 mL	4 mL

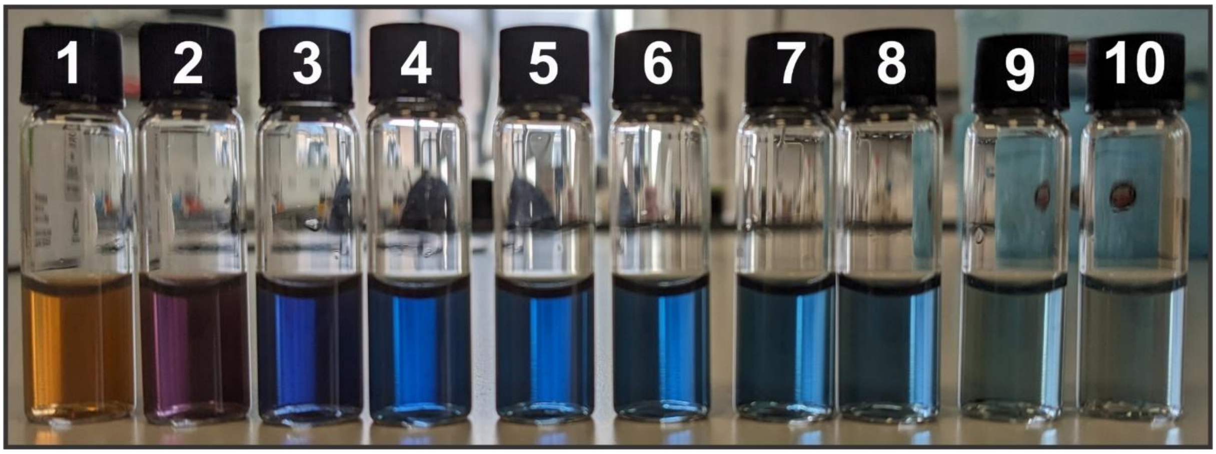

Figure S1. Optical images of the as made HGNs with different amounts of galvanic replacement. The numbers correspond to the sample numbers of Table S1.

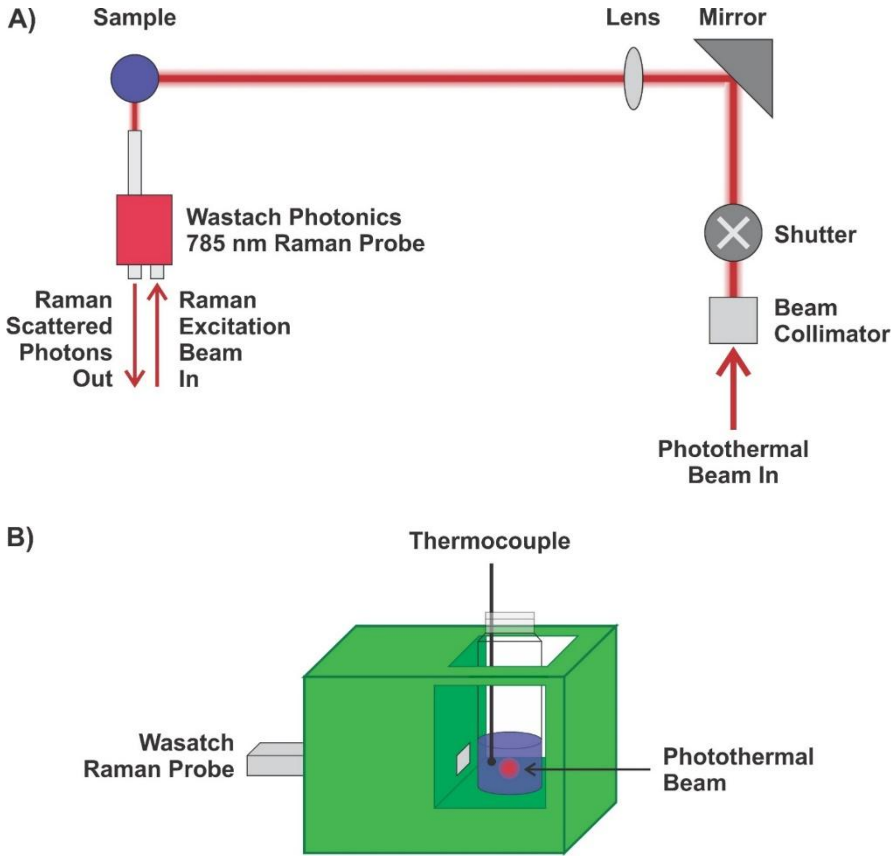

Figure S2. Representative diagrams of A) the optical paths, and B) how the sample, beam, and thermocouple are positioned with respect to the 3-D printed holder.

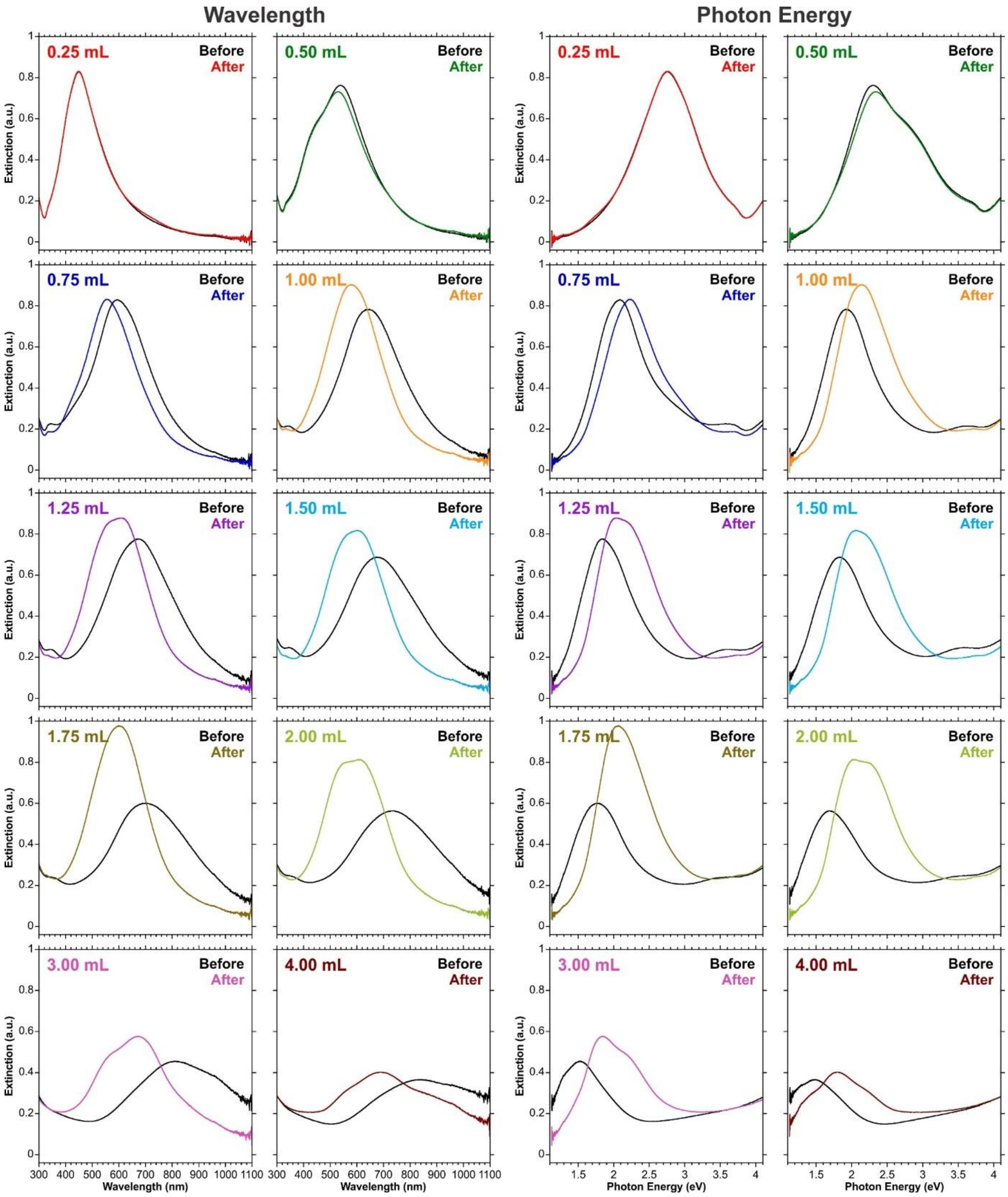

Figure S3. Comparison of extinction spectra of HGNs prepared with different volumes of K/Au (x mL of K/Au : 3 mL AgNPs : (10 – x) mL ddH_2O) before and after photothermal irradiation for 60 minutes. For clarity, both wavelength and photon energy scales are provided. These are the same samples from Figures 1A-C. Each spectrum is the average of 3 scans.

Table S2. Summary of the optical properties of the HGNs from Figure S1 before and after irradiation for 60 minutes.

Volume of K/Au (mL)	LSPR Before (nm)	LSPR After (nm)	ΔLSPR (eV)	Ext. @785 nm Before	Ext. @785 nm After	FWHM Before (eV)	FWHM After (eV)
0.25	450	448	0.0125	0.0690	0.0740	1.017	1.008
0.50	539	528	0.0479	0.136	0.132	1.265	1.270
0.75	595	554	0.154	0.290	0.173	0.9314	0.9865
1.00	644	581	0.209	0.440	0.223	0.8756	0.9648
1.25	672	605	0.204	0.540	0.260	0.9256	1.019
1.50	676	605	0.215	0.536	0.247	0.9814	1.022
1.75	698	599	0.294	0.533	0.272	1.008	0.9333
2.00	740	609	0.360	0.537	0.262	1.071	1.092
3.00	810	669	0.323	0.449	0.362	–	1.105
4.00	839	692	0.314	0.352	0.338	–	–

*FWHM is the full width half maximum of the LSPR. For the most red shifted samples, it was not possible to determine the full width half maximum (FWHM).

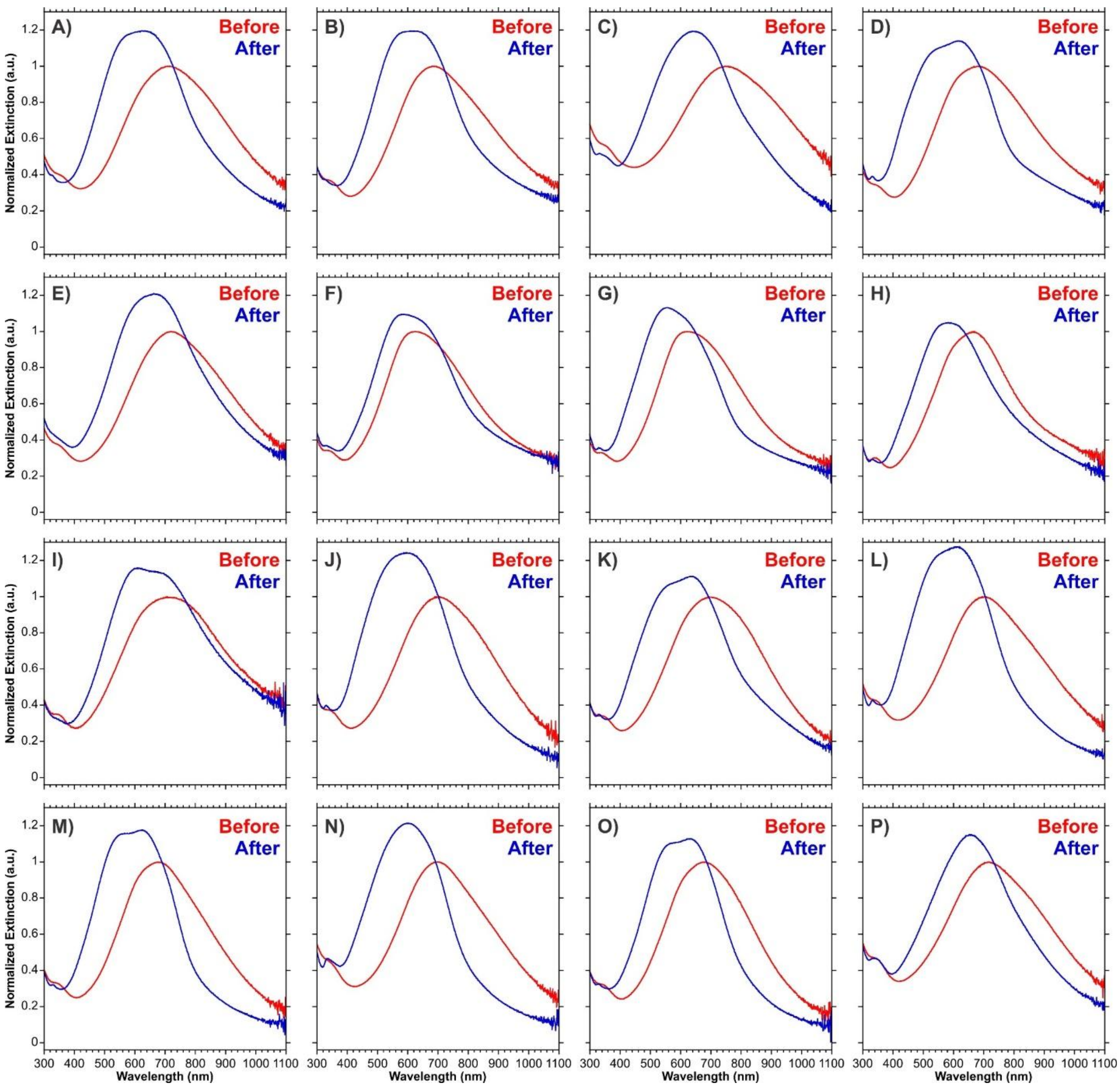

Figure S4. Normalized extinction spectra from 16 different experiments involving multiple batches of HGNs. The spectra are shown before and after irradiation, with the spectra normalized to the maximum extinction taken from the non-irradiated (Before) sample. A-G) were from experiments performed by author JG, and H-P) by author GQW.

Table S3. Summary of the optical properties of the HGNs from Figure S4 before and after photothermal irradiation.

**Panel from Fig. S4**	**LSPR Before (nm)**	**LSPR After (nm)**	**ΔLSPR (nm)**	**ΔLSPR (eV)**	**FWHM Before (eV)**	**FWHM After (eV)**	**ΔFWHM (eV)**
A)	714	633	81	0.222	1.130	1.273	0.143
B)	684	630	54	0.155	1.106	1.233	0.127
C)	747	637	110	0.287	1.244	1.309	0.065
D)	685	615	70	0.206	1.167	1.428	0.261
E)	720	663	57	0.148	1.058	1.169	0.111
F)	624	580	44	0.151	1.154	1.249	0.095
G)	621	557	64	0.229	1.131	1.342	0.211
H)	666	583	83	0.265	1.077	1.313	0.236
I)	706	608	98	0.283	1.208	1.298	0.090
J)	696	630	66	0.187	1.065	1.301	0.236
K)	693	642	51	0.142	1.062	1.357	0.295
L)	708	621	87	0.245	1.079	1.310	0.231
M)	677	627	50	0.146	1.017	1.195	0.178
N)	702	602	100	0.293	1.016	1.239	0.223
O)	679	630	49	0.142	1.006	1.149	0.143
P)	716	654	62	0.164	1.109	1.168	0.059
**Average**			70	0.20			0.17
**St. Dev.**			20	0.06			0.07

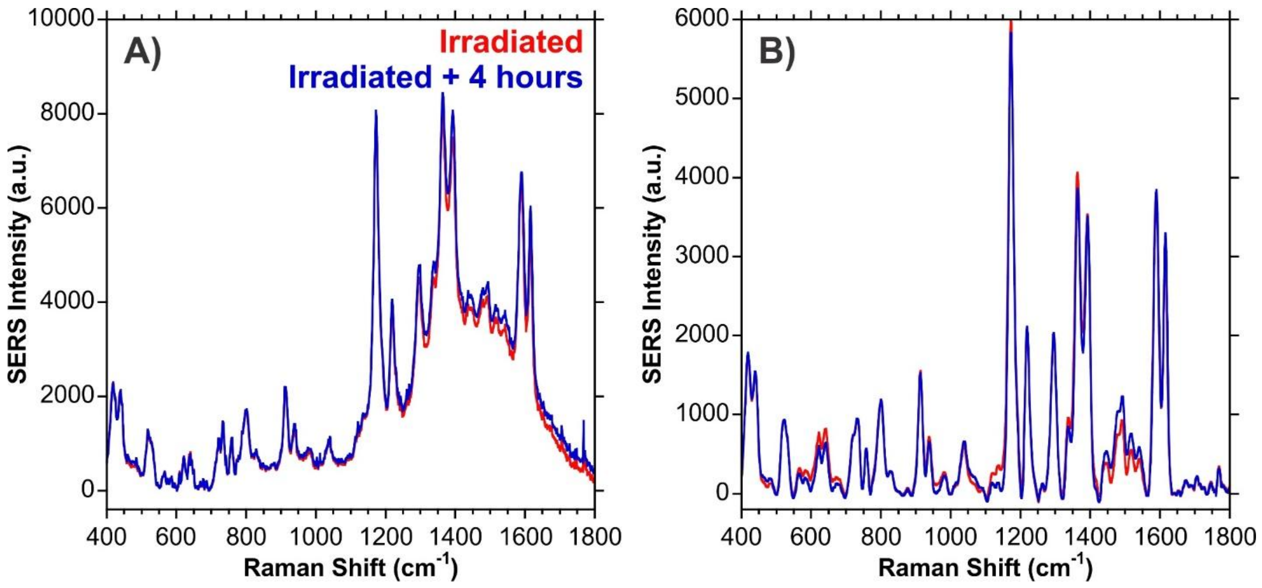

Figure S5. SERS spectra of malachite green isothiocyanate (MGITC) functionalized HGNs that were irradiated for 90 minutes under 785 nm photothermal heating, and the SERS spectra taken 4 hours after photothermal heating once the sample had cooled to room temperature. A) Spectra without having had a baseline correction applied, and B) after baseline correction. All spectra are the average of 10 spectra.

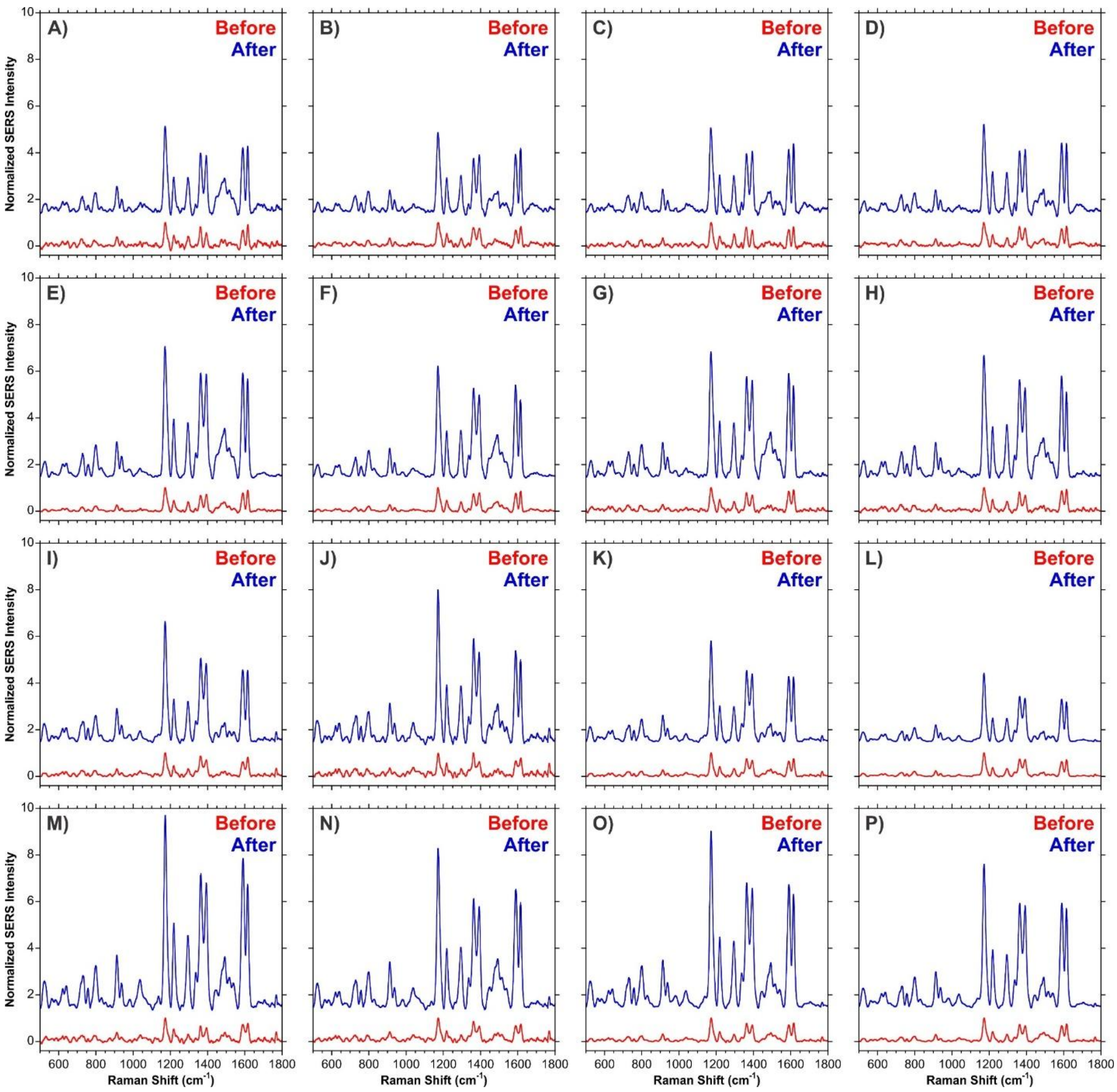

Figure S6. Baseline corrected and normalized SERS spectra from 16 different experiments involving multiple batches of HGNs functionalized with malachite green isothiocyanate (MGITC). The spectra are shown before and after irradiation, with the spectra normalized to the maximum SERS intensity taken from the non-irradiated (Before) sample. A-H) were from experiments performed by author JG, and I-P) by author GQW. Spectra have been offset for clarity.

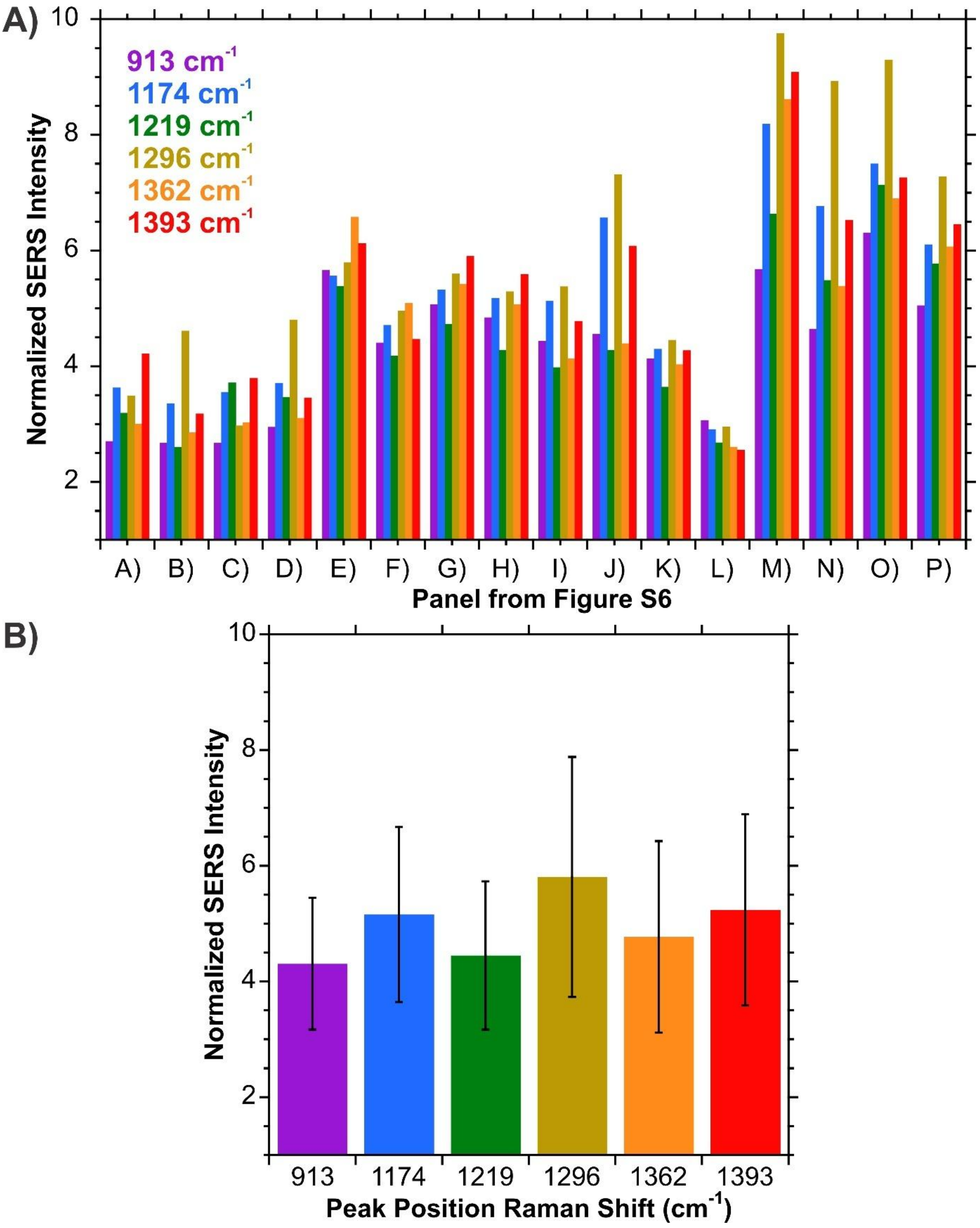

Figure S7. A) Normalized SERS intensities for six vibrational modes of MGITC obtained from the spectra of Figure S5 showing how the signal increases after irradiation vary. B) Average normalized SERS intensities for the six vibrational modes of interest. Error bars correspond to the standard deviation.

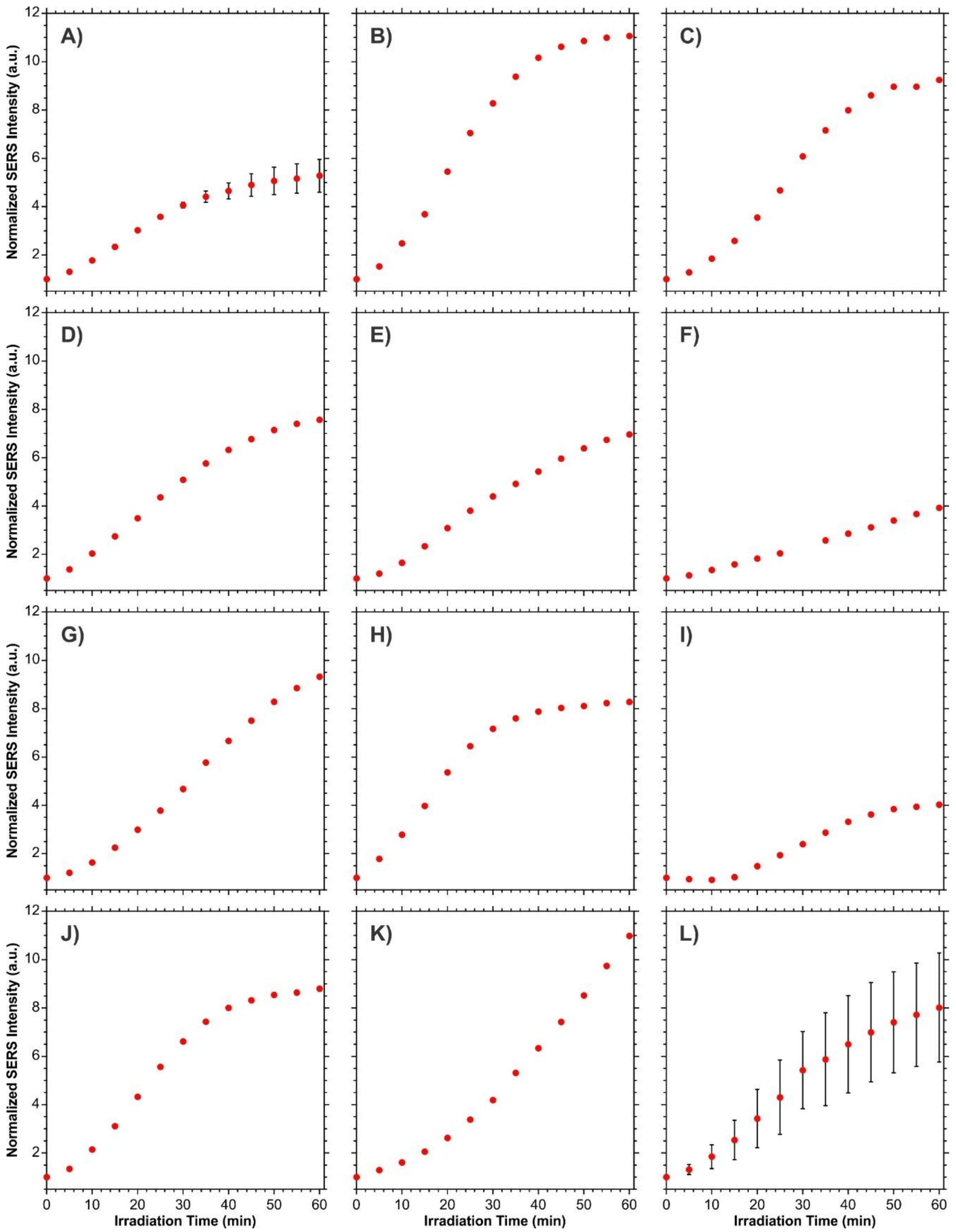

Figure S8. Temporal analysis for the variation of the SERS intensity of the 1174 cm^{-1} peak of MGITC under photothermal irradiation. A) Average and standard deviation of four runs of the same batch of AgHGNs. B-K) Various batches of AgHGNs. L) Average and standard deviation for the results of B-K).

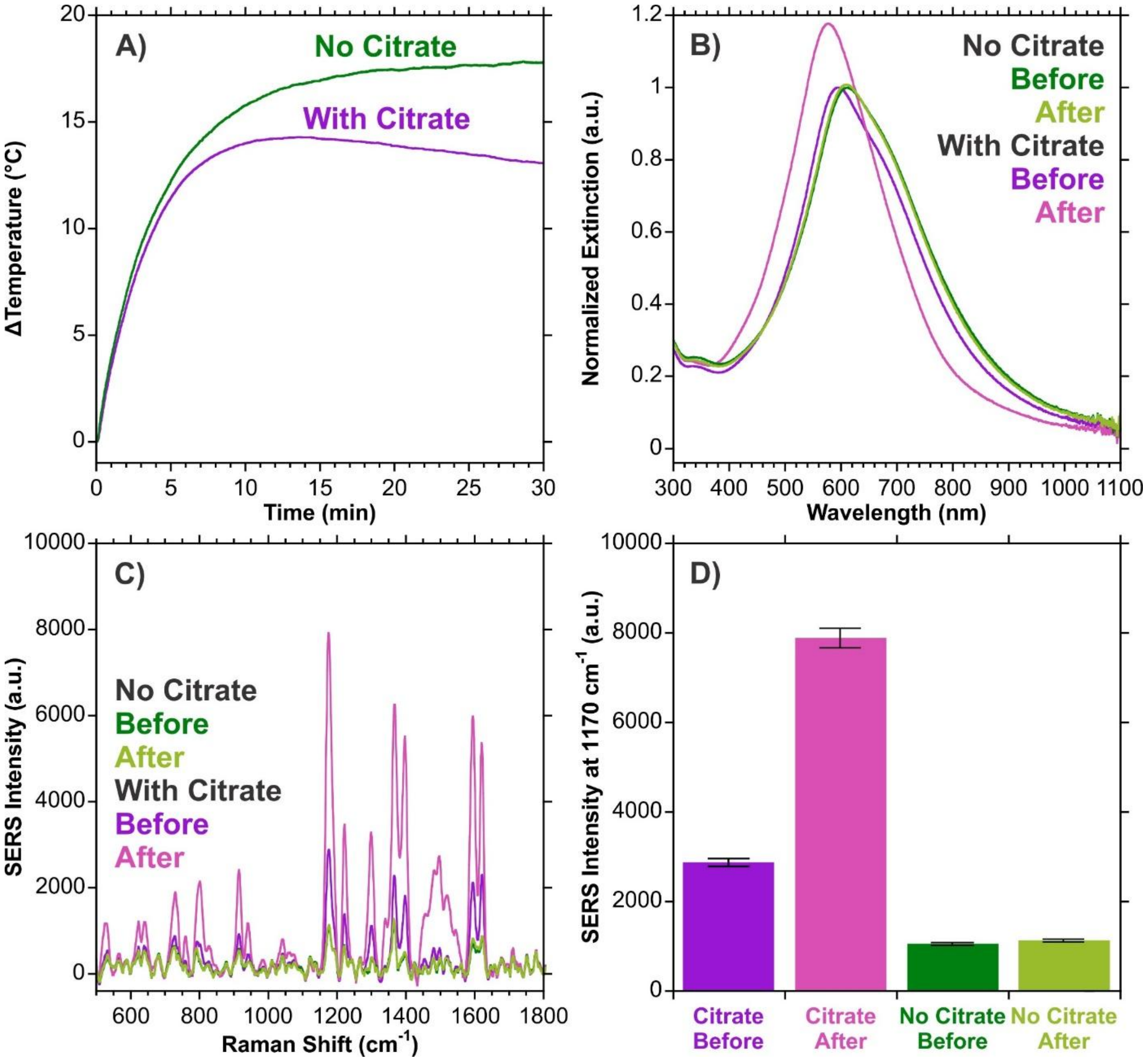

Figure S9. Triplicate comparison of HGNs prepared in the absence or presence of citrate before and after photothermal irradiation for 30 minutes. A) Bulk temperature profiles during photothermal irradiation, B) extinction spectra, C) SERS spectra of samples functionalized with MGITC, and D) average absolute SERS intensity of the 1170 cm-1 vibrational mode for samples functionalized with MGITC. All plots are the average of 3 replicate experiments. The errors in D) correspond to one standard deviation.

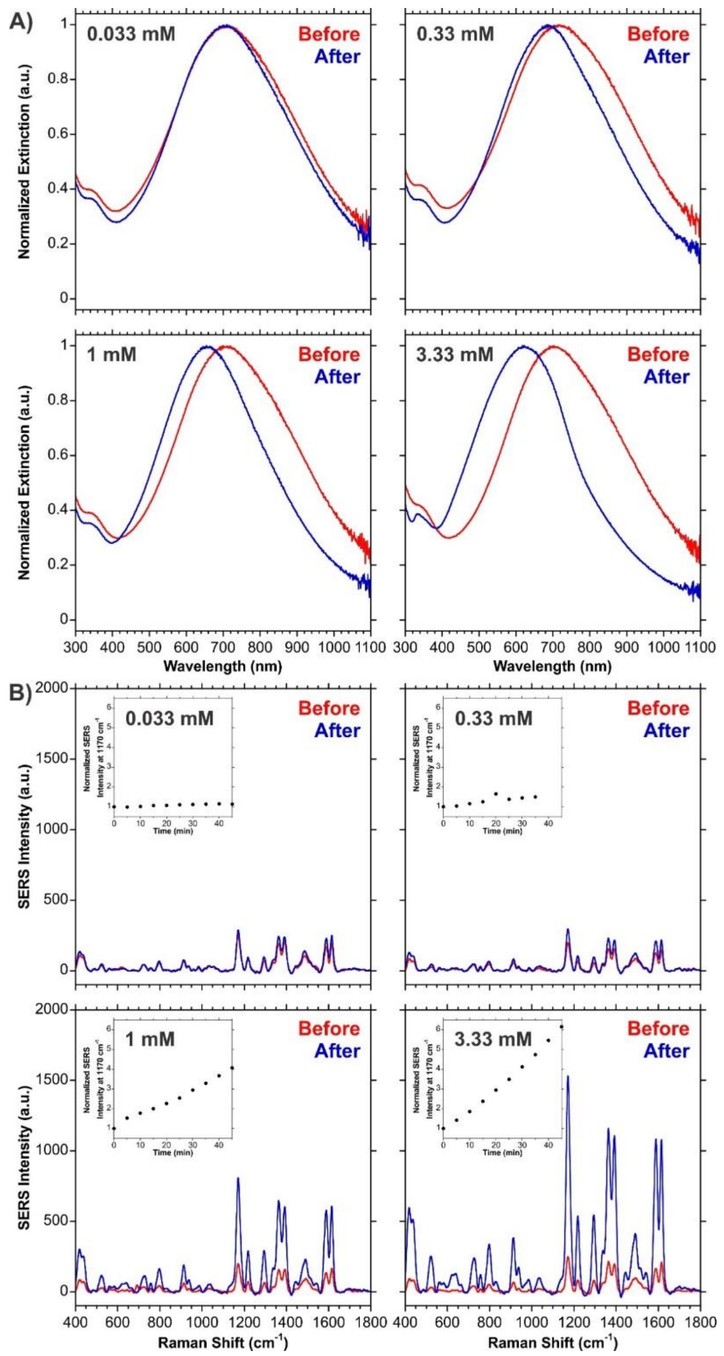

Figure S10. A) Normalized extinction spectra of HGNs suspensions in different concentrations of citrate before and after photothermally irradiated for 45 minutes. B) SERS spectra of MGITC functionalized HGNs samples before and after irradiation. Included as insets is the variation in the normalized SERS intensity at 1170 cm^{-1} as the samples are photothermally irradiated.

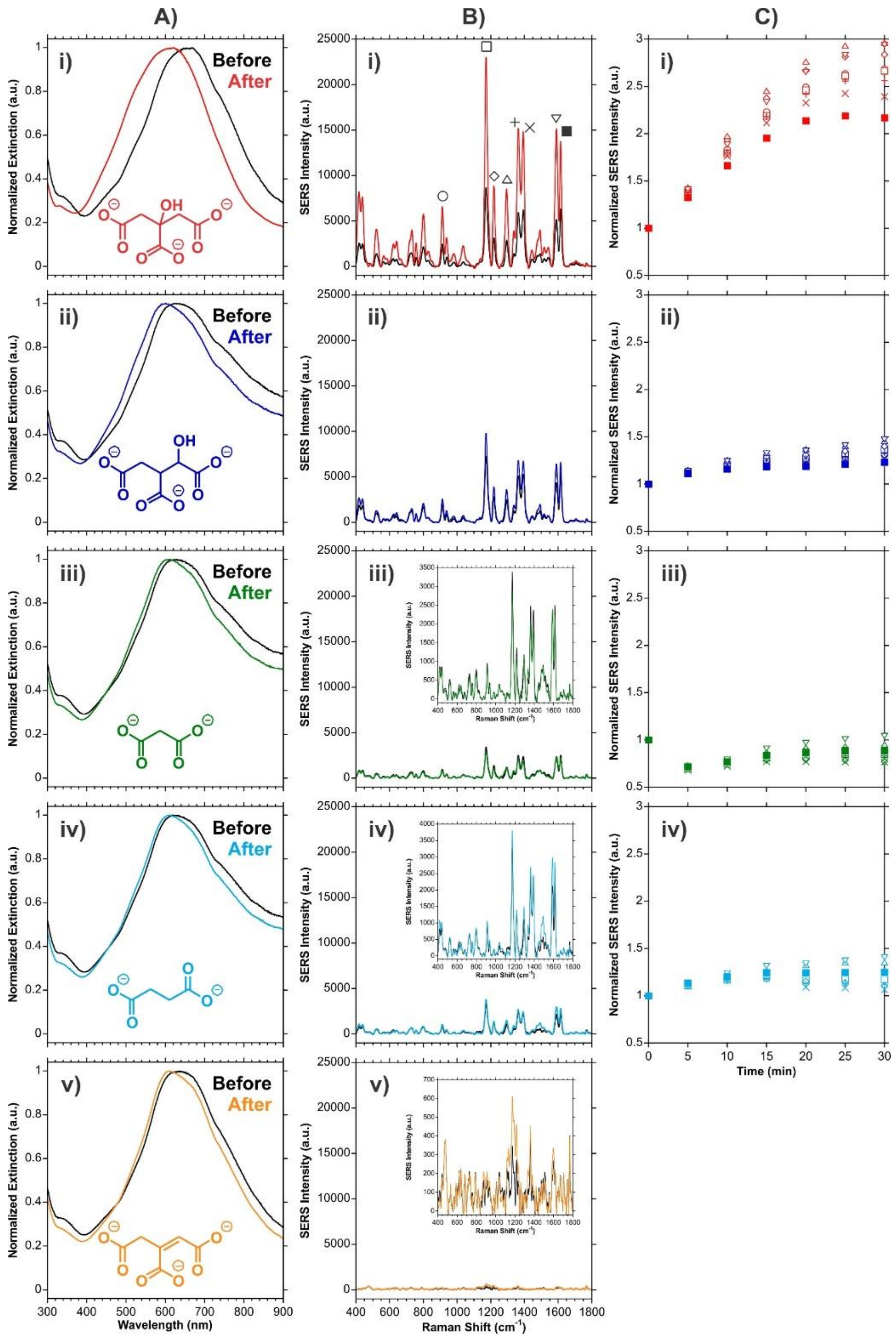

Figure S11. Comparison of HGNs stabilized in the presence of different ligands before and after 30 minutes of photothermal irradiation, A) normalized extinction spectra, B) SERS spectra of samples functionalized with MGITC, and C) temporal profiles for the some of the MGITC peaks normalized to the intensity prior to irradiation. The ligands evaluated are: i) citrate, ii) isocitrate, iii) malonate, iv) succinate, v) cis-aconitate. The structures for the molecules are included with the extinction spectra. For iii, iv, and v) SERS insets are included to better show the weak signals. It was not possible to perform the temporal analysis for the SERS intensity of cis-aconitate as the SERS spectrum of MGITC was not discernable.

Table S4. Summary of the optical properties of the HGNs from Figure S11 corresponding to citrate-free and citrate-stabilized HGNs heated in an oil bath for 20 minutes.

	**Citrate-Free**			**Citrate-Stabilized**		
**Oil Bath Temperature (°C)**	**LSPR Before (nm)**	**LSPR After (nm)**	**ΔLSPR (eV)**	**LSPR Before (nm)**	**LSPR After (nm)**	**ΔLSPR (eV)**
40	603	599	0.0137	609	601	0.0271
50	603	603	0	609	602	0.0237
60	603	602	0.00342	609	577	0.113
70	603	599	0.0137	609	554	0.202
80	603	598	0.0172	609	554	0.202
90	603	583	0.0705	609	555	0.198

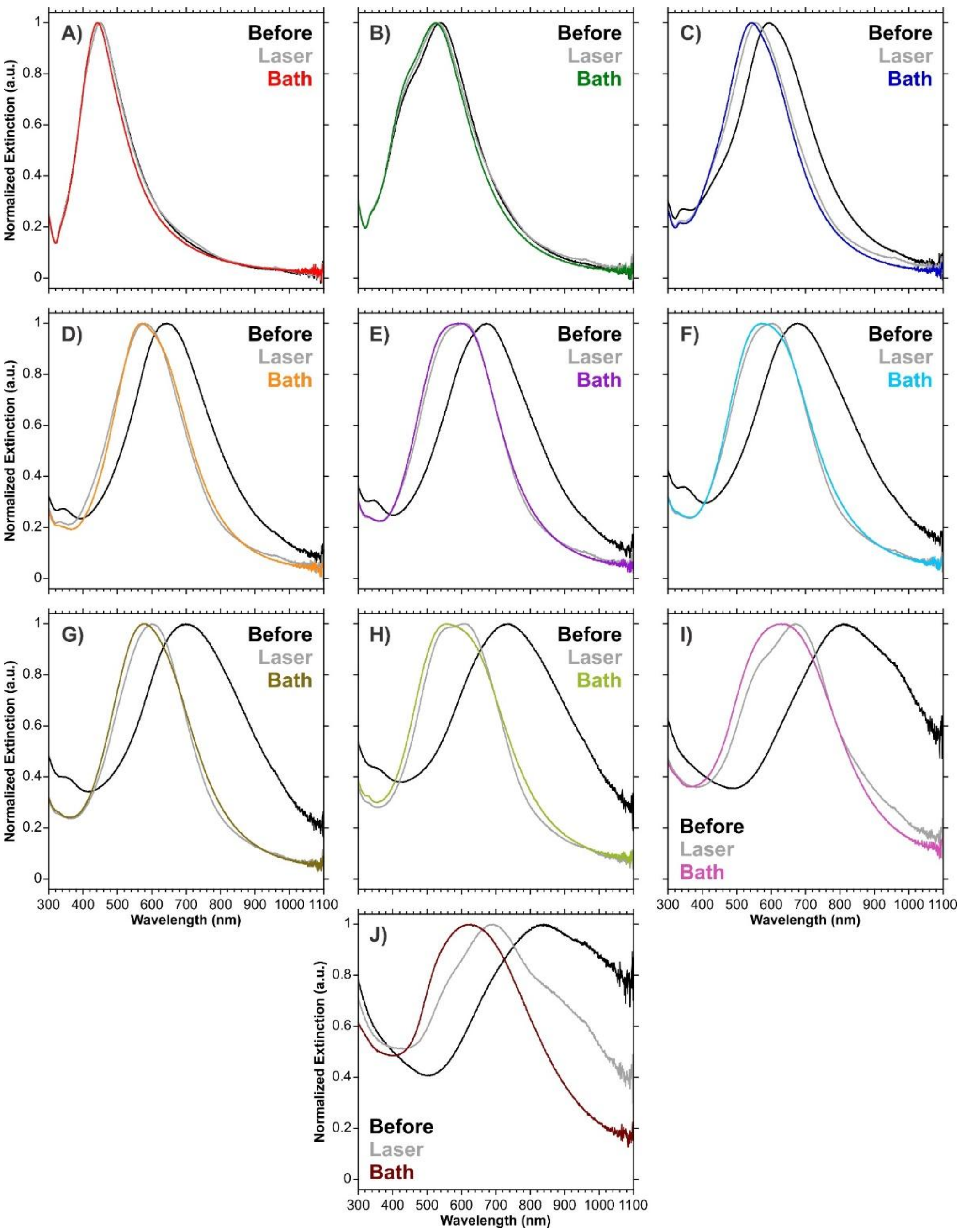

Figure S12. Normalized extinction spectra of HGNs prepared with different volumes of K/Au (3 mL AgNPs: (10 – x) mL ddH_2O: x mL of K/Au) before exposure, after laser exposure for 60 minutes, after heating in an oil bath at 70 °C for 20 minutes. A) 0.25, B) 0.50, C) 0.75, D) 1.00, E) 1.25, F) 1.50, G) 1.75, H) 2.00, I) 3.00, and J) 4.00 mL. Each spectrum is the average of 3 scans.

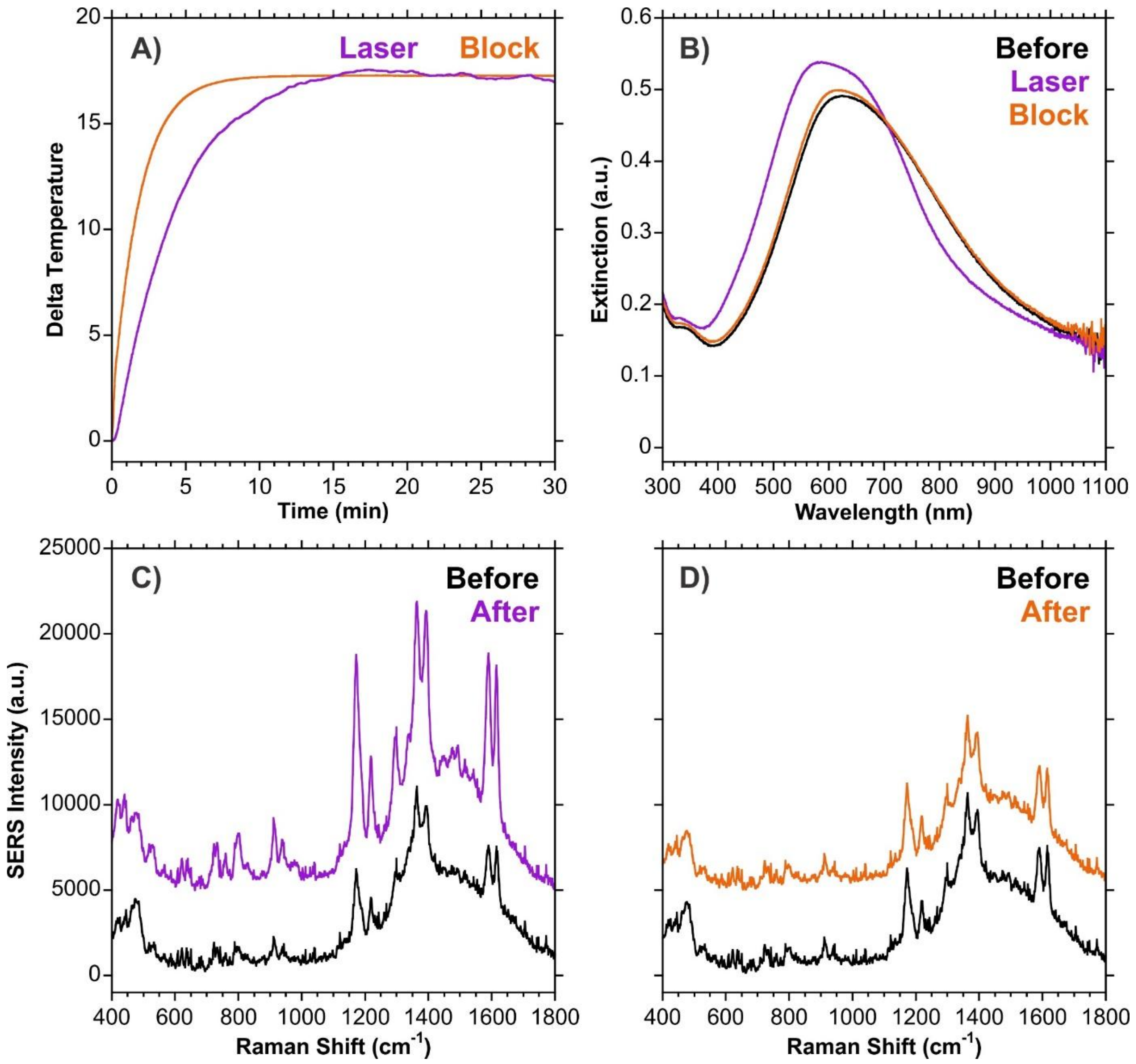

Figure S13. A) Bulk heating profiles of HGNs using a 785 nm laser (purple) and a heat block set at 38°C (orange). B) Extinction spectra of the HGNs before (black) and after being laser or block heated. SERS spectra of MGITC functionalized HGNs before and after C) laser irradiation, and D) block heating. SERS spectra have been offset for clarity.

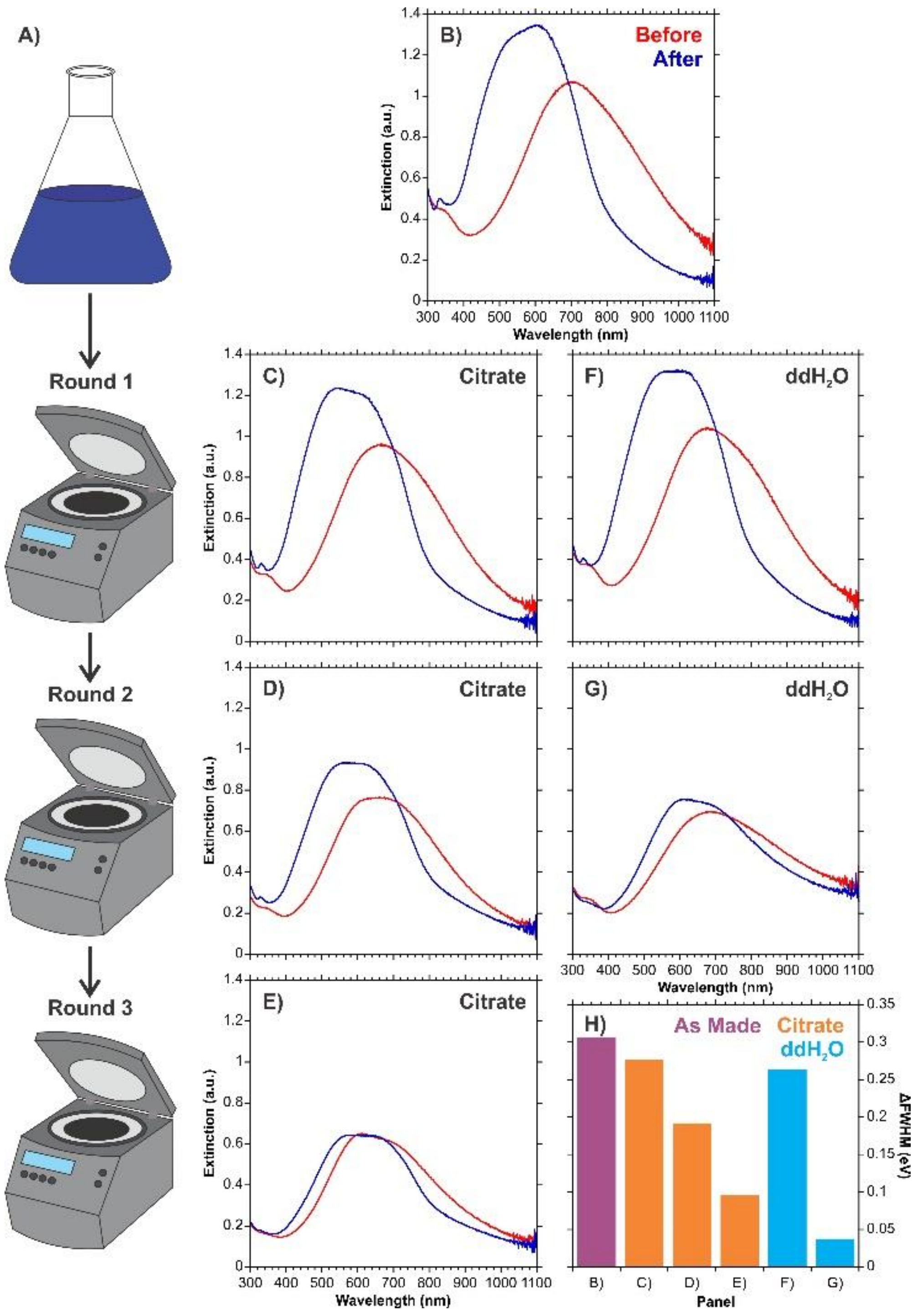

Figure S14. A) Schematic representation of the experiment whereby the as made AgHGNs underwent repeated rounds of centrifugation. B) Extinction spectra of the as made AgHGNs before and after irradiation. C-G) Extinction spectra for aliquots of AgHGNs taken after each round of centrifugation before and after irradiation. Samples were redispersed in either C-E) 3 mM citrate, or F-G) ddH2O. H) Change in the full width half maximum (FWHM) of the irradiated samples compared to the un-irradiated samples.

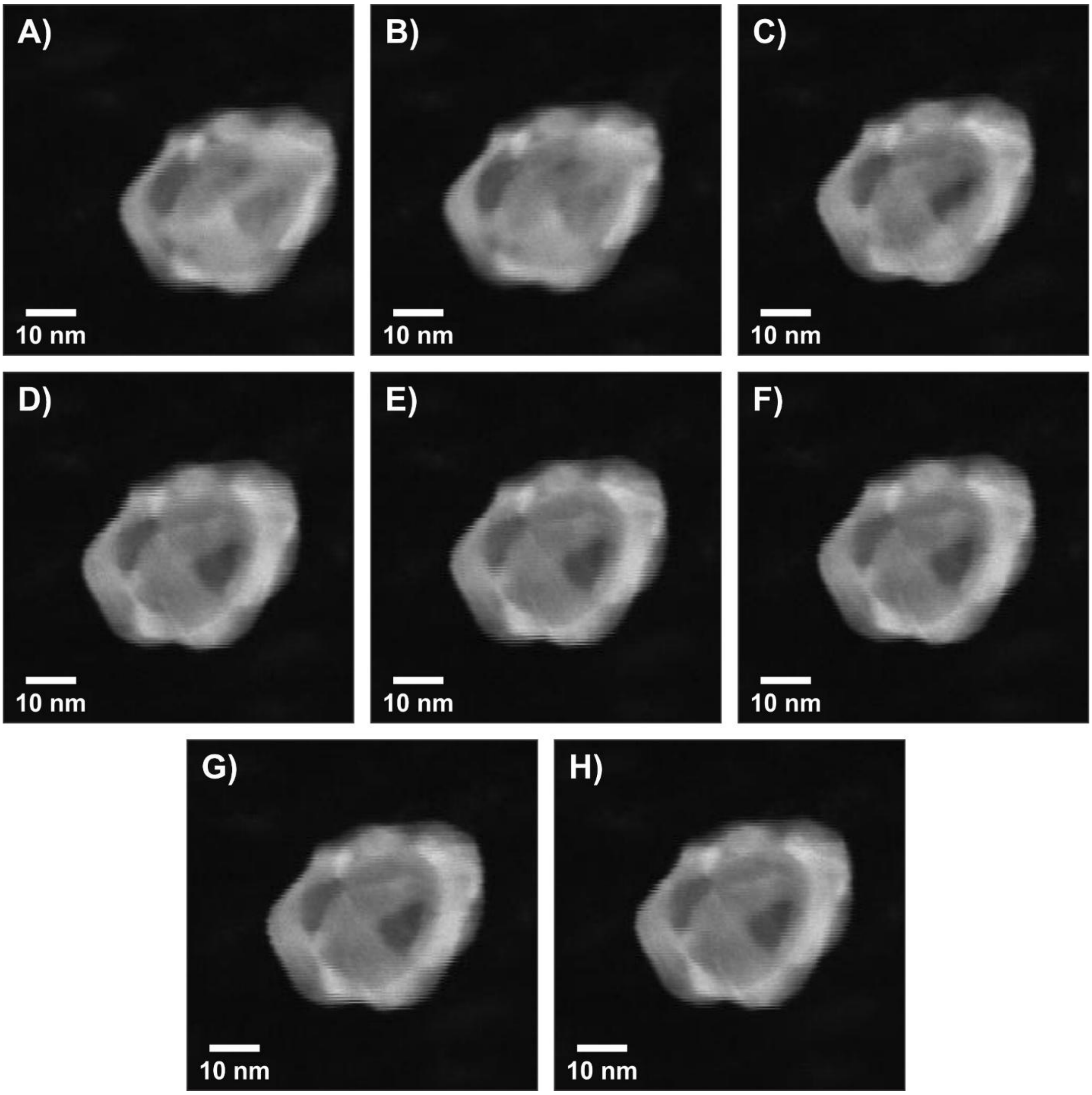

Figure S15. High-angle annular dark field scanning transmission electron microscopy (HAADF-STEM) images of an AgHGN as it is being imaged. The core of the AgHGN undergoes morphological changes over time. These images correspond to time points every second from the video of Supplementary Video 1.

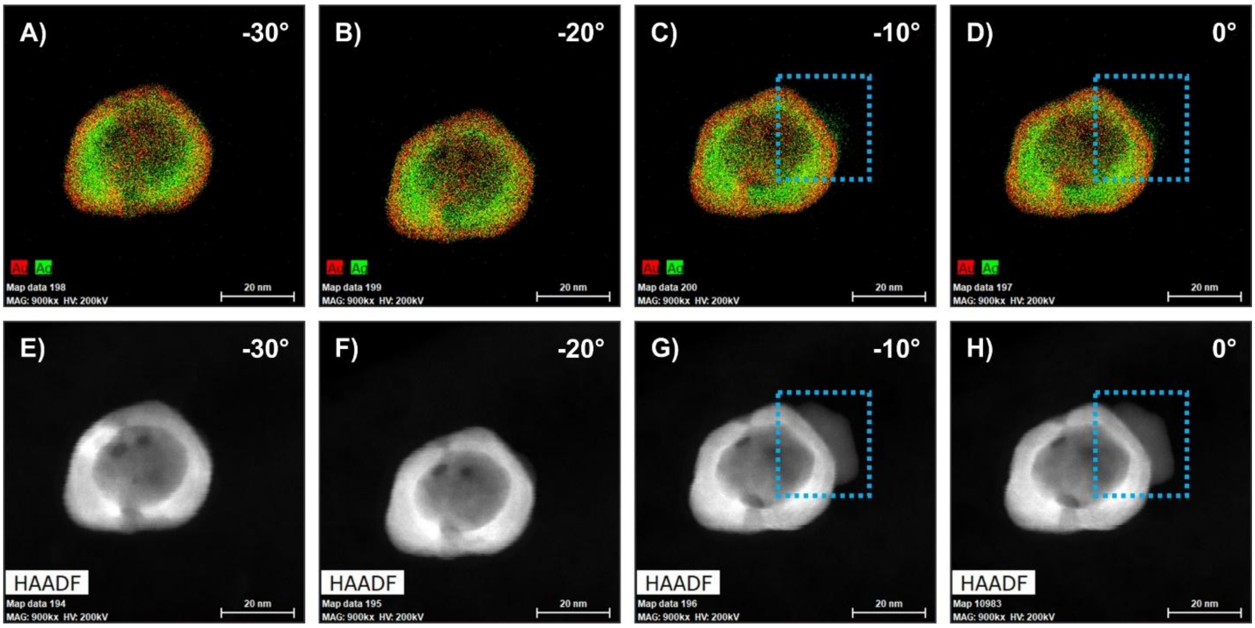

Figure S16. A-D) Energy dispersive x-ray (EDX) images of an AgHGN acquired in correlation with E-H) different HAADF-STEM images. The boxes of C, D, G, and H indicate areas where the presence of Ag was noticed during the measurements.

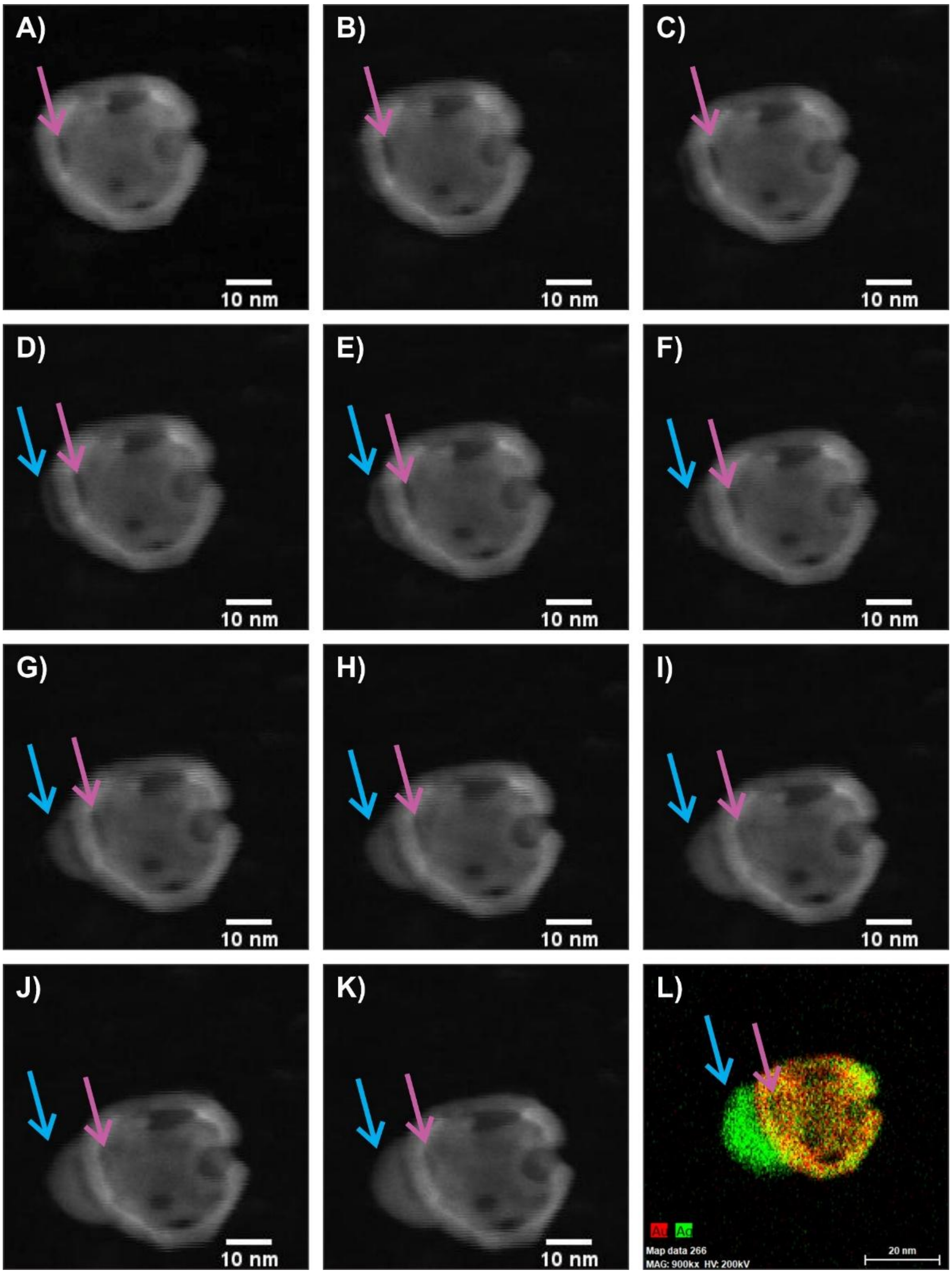

Figure S17. A-K) HAADF-STEM images of an AgHGN taken under constant exposure. L) EDX image taken at the end of the measurement. The light purple arrow indicates the presumed nucleation point for the silver growths indicated by the blue arrow. The HAADF images correspond to still images taken every 5 seconds from Supplementary Video 2.

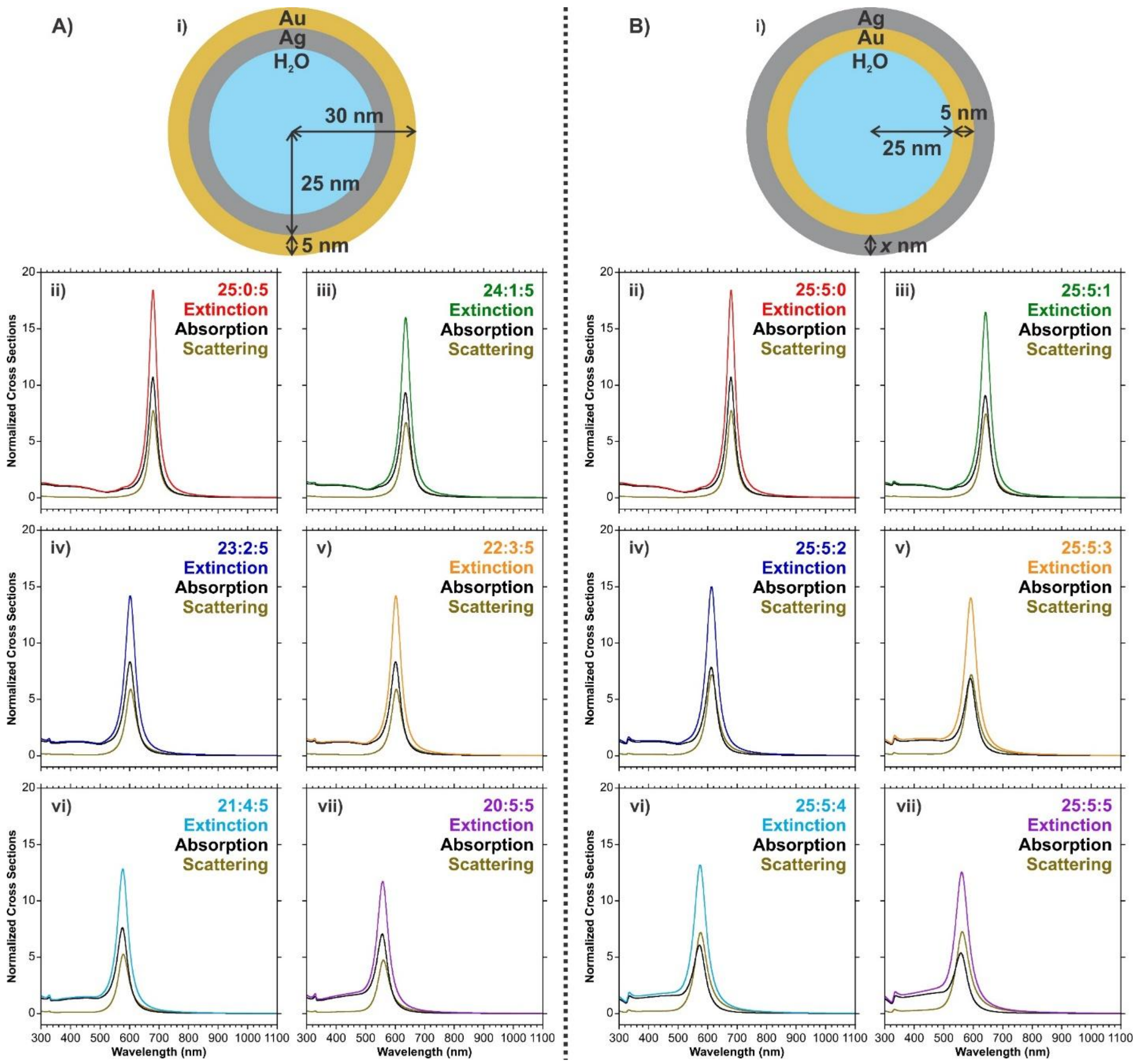

Figure S18. Comparison between the formation of a silver shell around the A) interior, and B) exterior of an HGN. i) Diagrams of the set-up considering a fixed total radius for the interior growth, and a fixed inner radius for exterior growth. ii-vii) Theoretical extinction, absorption, and scattering cross-sections determined using a Mie Theory calculator for different dimensions for the radius of the H_2O core: Ag shell thickness: Au shell thickness.[1] The refractive index of the hollow interior and surrounding media was set to 1.33, with the optical constants of silver and gold derived from the literature.[2]

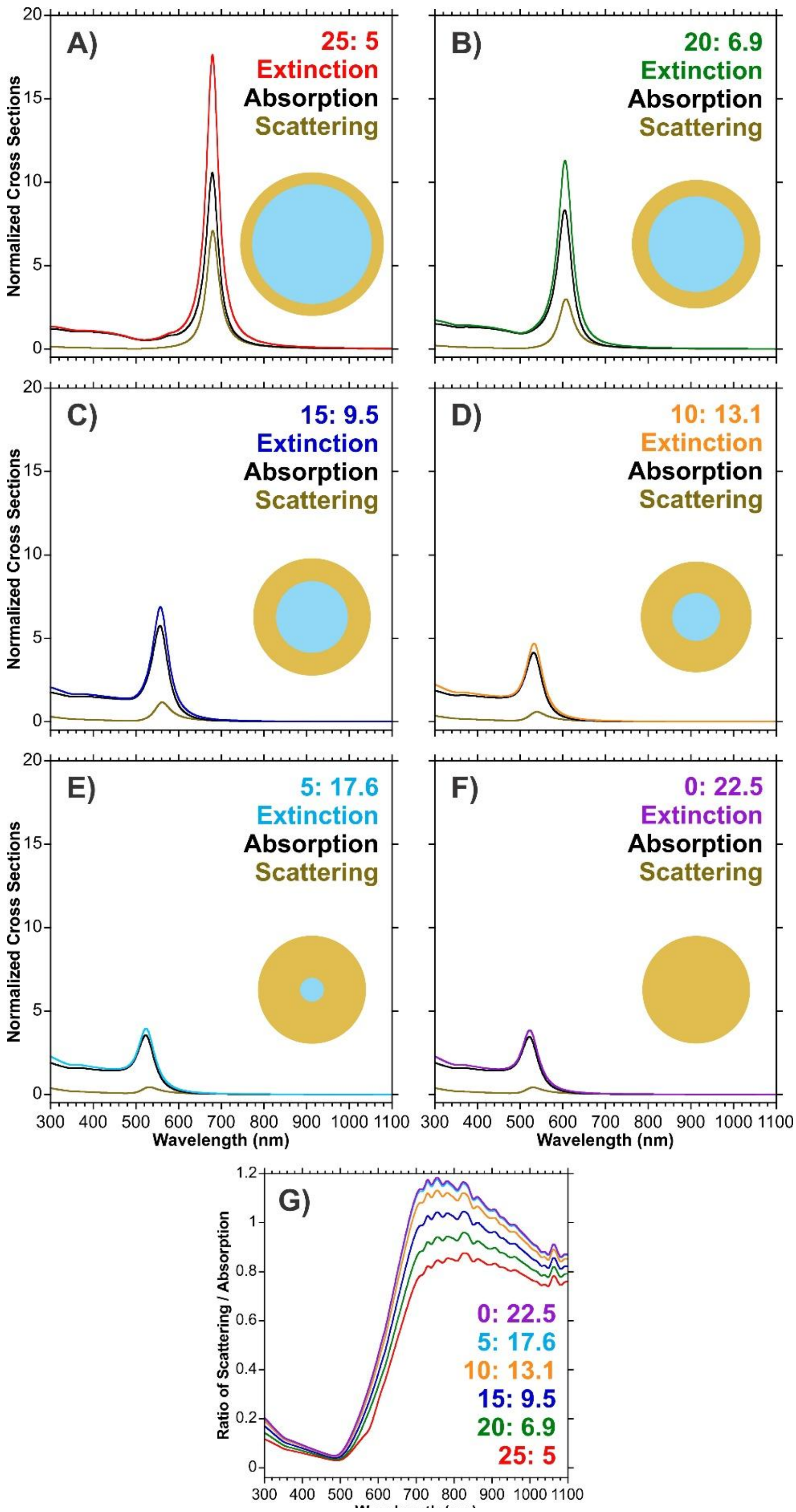

Figure S19. A-F) Theoretical extinction, absorption, and scattering cross-sections determined using a Mie Theory calculator for different dimensions for the radius of the H_2O core: Au shell thickness. Here, the total volume of gold is kept constant. G) Ratios of the calculated scattering cross-section to absorption cross-section.

Calculation of Ag Content

Moles of Ag = mass of $AgNO_3$ / molar mass of $AgNO_3$

Moles of Ag = 0.0223 g / 169.87 g/mol = 1.3×10^{-4} mol

Total Volume of AgNPs = Volume of NaOH + Volume of Hydroxylamine Hydrochloride + Volume of $AgNO_3$

Total Volume of AgNPs = 120 mL + 1.33 mL + 13.3 mL = 134.63 mL = 135 mL

Volume of AgNPs used = 3 mL

Moles of Ag in 3 mL = (1.3×10^{-4} mol) × 3 mL / 135 mL = 2.9×10^{-6} mol

Volume of HGNs = Volume of AgNPs + Volume of ddH2O + Volume of K/Au

Volume of HGNs = 3 mL + 6 mL + 1 mL = 10 mL

Concentration of Ag = 2.9×10^{-6} mol / 0.010 L = 2.9×10^{-4} M = ~ 0.3 mM

References

[1] K. Ladutenko; U. Pal; A. Rivera; O. Peña-Rodríguez, "Mie calculation of electromagnetic near-field for a multilayered sphere," *Comput. Phys. Commun.* **2017**, *214*, 225.

[2] K.M. McPeak; S.V. Jayanti; S.J.P. Kress; S. Meyer; S. Iotti; A. Rossinelli; D.J. Norris, "Plasmonic films can easily be better: Rules and recipes," *ACS Photonics* **2015**, 2, 326.
